# LOF variants identifying candidate genes of laterality defects patients with congenital heart disease

**DOI:** 10.1371/journal.pgen.1010530

**Published:** 2022-12-02

**Authors:** Sijie Liu, Wei Wei, Pengcheng Wang, Chunjie Liu, Xuechao Jiang, Tingting Li, Fen Li, Yurong Wu, Sun Chen, Kun Sun, Rang Xu

**Affiliations:** 1 Department of Pediatric Cardiology, Xinhua Hospital, School of Medicine, Shanghai Jiao Tong University, Shanghai, China; 2 Scientific Research Center, Xinhua Hospital, School of Medicine, Shanghai Jiao Tong University, Shanghai, China; 3 Department of Cardiology, Shanghai Children’s Medical Center, School of Medicine, Shanghai Jiao Tong University, Shanghai, China; University of Pennsylvania, UNITED STATES

## Abstract

Defects in laterality pattern can result in abnormal positioning of the internal organs during the early stages of embryogenesis, as manifested in heterotaxy syndrome and situs inversus, while laterality defects account for 3~7% of all congenital heart defects (CHDs). However, the pathogenic mechanism underlying most laterality defects remains unknown. In this study, we recruited 70 laterality defect patients with CHDs to identify candidate disease genes by exome sequencing. We then evaluated rare, loss-of-function (LOF) variants, identifying candidates by referring to previous literature. We chose *TRIP11*, *DNHD1*, *CFAP74*, and *EGR4* as candidates from 776 LOF variants that met the initial screening criteria. After the variants-to-gene mapping, we performed function research on these candidate genes. The expression patterns and functions of these four candidate genes were studied by whole-mount in situ hybridization, gene knockdown, and gene rescue methods in zebrafish models. Among the four genes, *trip11*, *dnhd1*, and *cfap74* morphant zebrafish displayed abnormalities in both cardiac looping and expression patterns of early signaling molecules, suggesting that these genes play important roles in the establishment of laterality patterns. Furthermore, we performed immunostaining and high-speed cilia video microscopy to investigate Kupffer’s vesicle organogenesis and ciliogenesis of morphant zebrafish. Impairments of Kupffer’s vesicle organogenesis or ciliogenesis were found in *trip11*, *dnhd1*, and *cfap74* morphant zebrafish, which revealed the possible pathogenic mechanism of their LOF variants in laterality defects. These results highlight the importance of rare, LOF variants in identifying disease-related genes and identifying new roles for *TRIP11*, *DNHD1*, and *CFAP74* in left-right patterning. Additionally, these findings are consistent with the complex genetics of laterality defects.

## Author summary

Defects in laterality pattern can result in abnormal positioning of the internal organs during the early stages of embryogenesis. Patients with laterality anomalies complicated by CHD have higher mortality as compared to their CHD peers without laterality anomalies. However, the pathogenic mechanism underlying most laterality defects remains unknown. In this study, we recruited 70 laterality defects patients with CHD to identify candidate disease genes by exome sequencing. We then evaluated rare, loss-of-function variants, identifying *TRIP11*, *DNHD1*, *CFAP74*, and *EGR4* as candidates. The expression patterns and functions of these four candidate genes were studied in zebrafish models. Among the four genes, *trip11*, *dnhd1*, and *cfap74* morphant zebrafish displayed abnormalities in both cardiac looping and expression patterns of early signaling pathways. In addition, impairments of Kupffer’s vesicle organogenesis or ciliogenesis were found in *trip11*, *dnhd1*, and *cfap74* morphant zebrafish. These results highlight the importance of rare, loss-of-function variants in identifying disease-related genes and display the new roles for *TRIP11*, *DNHD1*, and *CFAP74* in left-right patterning. Variants in *TRIP11* and *CFAP74* are identified in laterality defect patients for the first time.

## Introduction

Laterality defects are serious congenital malposition complexes characterized by defects of embryonic left-right (LR) patterning. They present as a range of developmental disorders, including situs inversus (SI) and heterotaxy (HTX) syndrome [1]. SI is characterized by complete, mirror-image reversal of all asymmetrical structures, whereas HTX is defined as having at least one organ discordant along the left-right axis and is traditionally classified into two groups: left atrial isomerism and right atrial isomerism [2]. Laterality defects have an estimated global prevalence of 1/10,000 and account for approximately 3~7% of all congenital heart defects (CHDs) [3,4]. Patients with laterality anomalies complicated by CHD have higher mortality as compared to their CHD peers without laterality anomalies. A previous study showed that the postoperative mortality of CHD patients with heterotaxy after surgical treatments was 4.8%, while the mortality of CHD patients without heterotaxy was 2.4% [5]. Patients with heterotaxy can have a diverse range of complex cardiac anomalies spanning all lesion types, including double outlet right ventricle (DORV), atrioventricular canal defects (AVC), anomalous pulmonary venous connection (APVC), transposition of the great arteries (TGA), single atrium (SA) and single ventricle (SV), etc. [6].

The mechanism underlying LR patterning is highly conserved among distinct classes of vertebrates. At the early somite stage, the LR axis develops symmetrically. LR asymmetry is initiated by the directional rotation of cilia in a conserved ciliated organ, the LR organizer (LRO; also referred to as the “node” in mice and the “Kupffer’s vesicle” in zebrafish). Subsequently, asymmetry signals are transmitted to the left lateral plate mesoderm (LPM) to induce asymmetric expressions of genes, such as *Nodal*, left-right determination factor 2 (*Lefty2*), and paired-like homeodomain 2 (*pitx2*), and lead to LR asymmetric morphogenesis of the internal organs [2]. Several signaling pathways, such as Notch, Nodal, Hedgehog, Wnt, and transforming growth factor-beta 1 (TGF-1), which are involved in the formation of the LR axis, are conserved in all vertebrates. Previous studies have demonstrated an association between either ciliary disorders (defects of structure or function) or the dysfunction of early cell signaling during embryogenesis and laterality defects in different organisms [7–10]. In humans, mutations in the genes involved in ciliary formation (e.g., *DNAH5*, *NPHP4*) and pleiotropic signaling pathways (e.g., *NODAL*, *CFC1*, *ACVR2B*, *LEFTY2*, *ZIC3*) have been identified in patients with laterality defects [6,11–13]. However, the known mutations only account for <20% of cases with laterality defects; [6] the etiology of laterality defects for the majority of affected patients remains unknown.

Exome sequencing (ES) is an efficient strategy to selectively sequence genomic coding regions (exons) for the identification of variations associated with human disease phenotypes, including single nucleotide variations (SNVs) and indels. ES improves the ability to detect pathologic genetic variations in complex diseases [11]. Several studies utilizing ES have demonstrated that many diseases are related to SNVs or indels, such as CHD and hypophosphatasia with mental retardation syndrome [14–16]. In previous research, both Alexander H. Li and Shuzhang Liang screened candidate laterality defect-related genes by ES [11,17]. Moreover, *MMP21* and *SHROOM3* were identified in heterotaxy patients through ES and found responsible for left-right asymmetry by further experiments in animal models [3,18]. All the studies above suggest that ES can be used for analyzing the genetic factors of laterality defects.

In our study, we identified 39 genes with multiple loss-of-function (LOF) variants through ES screening in 70 unrelated patients with laterality defects. Among these candidate variants, we found four potential genes involved in either ciliary proteome formation and function or the Nodal signaling pathway. The downregulation of three out of the four genes identified (*trip11*, *dnhd1*, and *cfap74*) in zebrafish caused disorders in both cardiac looping and the expression patterns of nodal-responsive genes (*spaw*, *lefty2*, and *pitx2*). Furthermore, our results showed that knockdown of *trip11*, *dnhd1*, and *cfap*74 in zebrafish altered Kupffer’s vesicle organogenesis or ciliogenesis. To our knowledge, this is the first study to identify *TRIP11* and *CFAP74* as novel laterality defect-related genes involved in LR patterning in both humans and animals.

## Results

### Clinical data

A total of 70 unrelated Chinese patients with laterality defects were recruited for our study. All patients exhibited abnormal positioning of the internal organs and cardiac abnormalities without non-laterality-associated malformations or other syndromes. No family histories of any congenital malformations were noted in the patients’ medical records. Patient ages ranged from 4 days to 16 years; 46 patients were male and 24 were female. Detailed information about the cardiac and extracardiac congenital malformations is summarized in Table 1. Total/partial anomalous pulmonary venous connection (TAPVC/PAPVC) was observed in 18 patients, double outlet right ventricle (DORV) in 28 patients, complete/partial atrioventricular canal (CAVC/PAVC) in 28 patients, and pulmonary atresia/stenosis (PA/PS) was identified in 61 patients. Thirty-one patients had malposed or transposed great arteries (MGA/TGA).

**Table 1:** Cardiac and extracardiac abnormalities in the patients with laterality defects.

	Number of patients (%)
Sex	
Male	46 (65.7%)
Female	24 (34.3%)
Cardiac position	
Levocardia	21 (30%)
Dextrocardia	42 (60%)
Mesocardia	6 (10%)
Atrial arrangement	
Atrial situs inversus	23 (32.9%)
Isomerism of right atrial appendages	40 (57.1%)
Isomerism of left atrial appendages	5 (7.1%)
Ventricular arrangement	
Ventricular situs solitus	21 (30%)
Ventricular situs inversus	21 (30%)
Single ventricle	28 (40%)
Bronchi	
Bilateral right bronchi (short)	38 (54.3%)
Bilateral left bronchi (long)	6 (8.6%)
Bronchial inversus	26 (37.1%)
Spleen	
Polysplenia	5 (7.1%)
Asplenia	38 (54.3%)
Single right spleen	25 (35.7%)
Single left spleen	2 (2.9%)
Stomach	
Right-sided stomach	38 (54.3%)
Left-sided stomach	23 (32.9%)
Stomach centrally situated	6 (8.6%)
Unknown	3 (4.3%)
Liver	
Left-sided liver	23 (32.9%)
Right-sided liver	8 (11.4%)
Liver centrally situated	39 (55.7%)
Aortic arch	
Left aortic arch	32 (45.7%)
Right aortic arch	38 (54.3%)
SVC	
Right SVC	10 (14.3%)
Left SVC	35 (50%)
Bilateral SVC	25 (35.7%)
IVC	
Interrupted IVC, hemiazygos vein continuation	2 (2.9%)
Interrupted IVC, azygos vein continuation	5 (7.1%)
Relationship of IVC and descending aorta	
IVC right of spine and descending aorta left of spine	5 (7.1%)
IVC left of spine and descending aorta right of spine	19 (27.1%)
IVC and descending aorta same side	35 (50%)
IVC left of spine and descending aorta anterior of spine	2 (2.9%)
IVC anterior of spine and descending aorta left of spine	1 (1.4%)
IVC right of spine and descending aorta anterior of spine	1 (1.4%)

### Identification of candidate genes

**Fig 1:**
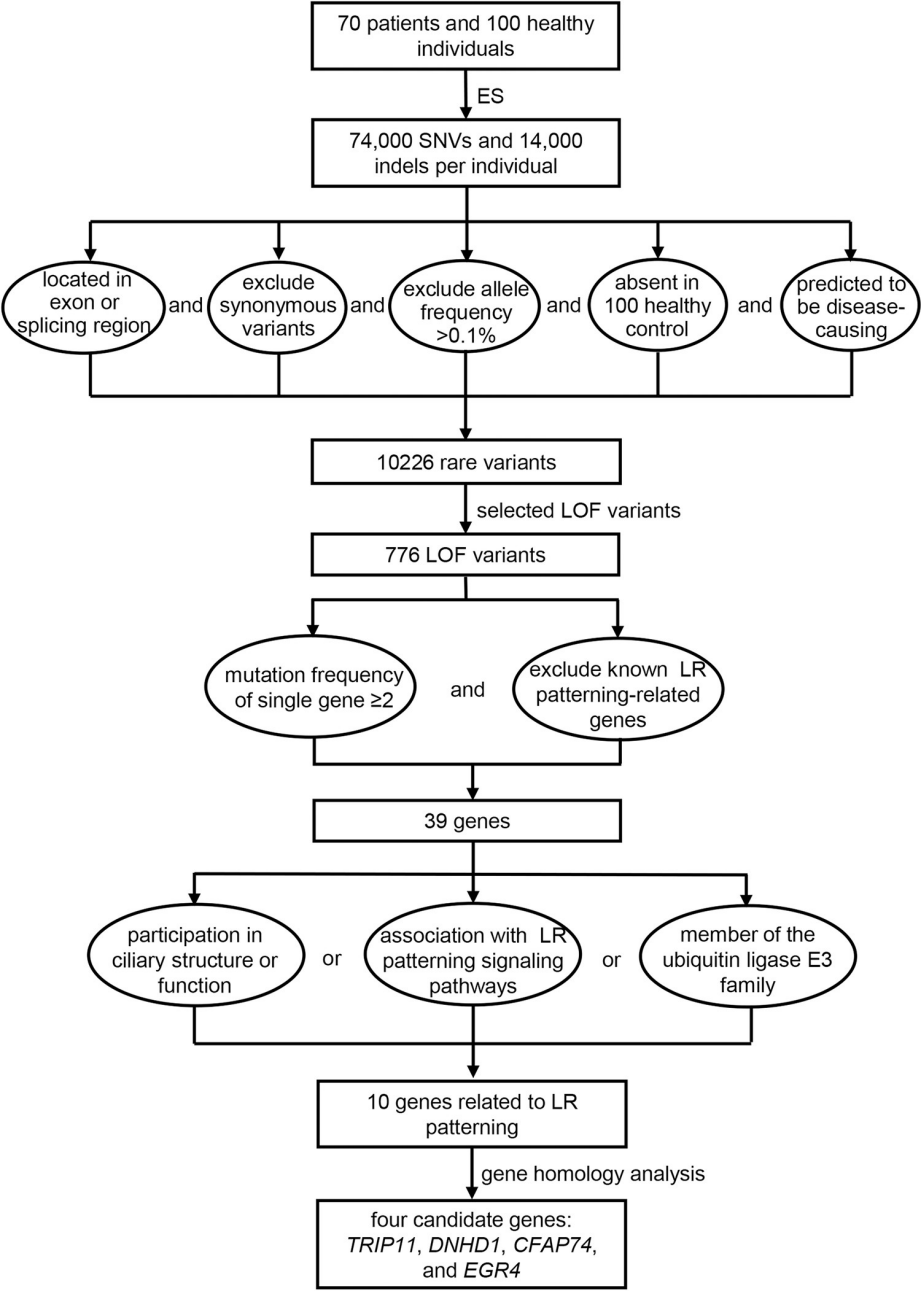
Schematic of the methodology applied to identify candidate genes. The results of ES performed on 70 patients were screened according to a series of criteria. 10226 rare variants were screened out. Among them, 776 LOF variants were selected for further analysis. 39 genes were identified from LOF variants for function and homology analysis. Finally, *TRIP11*, *DNHD1*, *CFAP74*, and *EGR4* were selected as candidate genes for further study.

To identify the genetic causes of the laterality defects, we performed ES on 70 patients and 100 healthy individuals. (Fig 1) The results revealed approximately 74,000 SNVs and 14,000 indels per individual. To identify potential disease-related genes in patients with laterality defects, variants were selected based on the following criteria: (1) located in exon or splicing region; (2) exclude synonymous variants; (3) exclude variants with allele frequency >0.1% in 1000 Genomes Project or ExAC; (4) absent in dataset of 100 healthy control individuals; (5) predicted to be disease-causing by at least one online program. According to these criteria, we identified up to 10226 potential variants, including both SNV and indel. To narrow the range of options, we selected LOF variants consisting of frameshift, nonsense, and splice-site variants, and further screened 776 candidate variants. (S1 Table)

While 39 genes were identified more than once in the 776 selected variants (Table 2), they did not include any known LR pattern-related genes (e.g., *ZIC3*, *CFC1*, *NKX2*.*5*, *GDF1*, *NODAL*, *LEFTY1*, *LEFTY2*, *ACVR2B*, *DNAH5*, *DNAH11*, *DNAI1*, *FOXH1*, *CRELD1*, and *GALNT11*). We then examined the functions of the 39 candidate genes to correlate the laterality defect phenotypes to specific genes. All candidate genes were identified according to the following criteria: (1) participation in ciliary structure or function; (2) association with LR axis formation-associated signaling pathways, including Notch, Nodal, TGF-β, Hedgehog, and Wnt; (3) member of the ubiquitin ligase E3 family. We then identified 10 genes (*C11orf94*, *C16orf71*, *C2orf71*, *CABS1*, *CFAP74*, *CUL7*, *DNHD1*, *EGR4*, *FSIP2*, and *TRIP11*) related to LR patterning. Among them, *C2orf71* was excluded, because a previous study conducted a morphological examination of the *C2orf71* morphants revealed no gross defects in the body axis in zebrafish [19]. Considering 5 out of these 10 genes without homologous genes in zebrafish (Table 2), we finally choose *TRIP11*, *DNHD1*, *CFAP74*, and *EGR4* as our candidate genes (S1 and S2 Figs). There were 10 LOF variants located in 4 candidate genes, including 1 splicing, 3 stop gain, and 6 frameshift variants (Table 3), which were detected in 10 patients, respectively (Table 4).

**Table 2:** The bioinformatics information on the LOF variants of candidate genes.

Chromosome	Gene	OMIM number	Component	Function	Homology (Human vs zebrafish)
5p14.1	ACOT12	614315	Acyl-CoA Thioesterase 12	fatty Acyl-CoA Biosynthesis and Metabolism	60.46%
9p13.3	ANKRD18B	618930	Ankyrin Repeat Domain 18B	nucleotide binding	12%
12q24.12	ATXN2	601517	Spinocerebellar Ataxia Type 2 Protein	negative regulator of endocytic EGFR internalization at the plasma membrane	62.24%
11p11.2	C11orf94	/	Chromosome 11 Open Reading Frame 94	Cornelia De Lange Syndrome 4 With or Without Midline Brain Defects	/
16p13.3	C16orf13	/	Chromosome 16 Open Reading Frame 1	Left Ventricular Noncompaction	58.78%
16p13.3	C16orf71	/	Chromosome 16 Open Reading Frame 71	an axonemal dynein regulator	/
1q32.1	C1orf116	611680	Chromosome 1 Open Reading Frame 116	putative androgen-specific receptor	33%
2p23.2	C2orf71	613425	Photoreceptor Cilium Actin Regulator	normal photoreceptor cell maintenance and vision	44.58%
4q13.3	CABS1	618600	Calcium Binding Protein, Spermatid Associated 1	calcium-binding protein	/
**1p36.33**	**CFAP74**	**/**	**Cilia and Flagella Associated Protein 74**	**part of the central apparatus of the cilium axoneme**	**48.02%**
19p13.3	CFD	134350	Complement Factor D	functions as an adipokine and complement activation	50.76%
2p13.3	CLEC4F	/	C-Type Lectin Domain Family 4 Member F	receptor with an affinity for galactose and fucose	17%
4p16.3	CRIPAK	610203	Cysteine-rich PAK1 inhibitor	negative regulator of PAK1	/
6p21.1	CUL7	609577	Cullin 7	an E3 ubiquitin-protein ligase complex	/
**11p15.4**	**DNHD1**	**617277**	**Dynein Heavy Chain Domain 1-Like Protein**	**microtubule motor activity**	**43.08%**
10q26.13	DMBT1	601969	Deleted In Malignant Brain Tumors 1	surfactant metabolism and Salivary secretion	53.98%
**2p13.2**	**EGR4**	**128992**	**Early Growth Response 4**	**transcriptional regulator**	**34%**
2p16.3	FSHR	136435	Follicle Stimulating Hormone Receptor	G protein-coupled receptor for follitropin	63.03%
2q32.1	FSIP2	615796	Fibrous Sheath Interacting Protein 2	play a role in spermatogenesis	/
19p13.3	FUT5	136835	Fucosyltransferase 5	fucosyltransferase activity	/
14q12	GZMH	116831	Granzyme H	cytotoxic chymotrypsin-like serine protease with preference for bulky	/
5q35.2	HK3	142570	Hexokinase 3	catalyzes the phosphorylation of hexose	/
1q21.3	HRNR	616293	Hornerin	component of the epidermal cornified cell envelopes	/
22q13.33	LMF2	/	Lipase Maturation Factor 2	involved in the maturation of specific proteins in the endoplasmic reticulum	61.53%
5p15.33	LRRC14B	/	Leucine-Rich Repeat Containing 14B	a member of the PRAME family	54.15%
19p13.2	MUC16	606154	Mucin 16, Cell Surface Associated	provide a protective, lubricating barrier against particles and infectious agents at mucosal surfaces	/
7q22.1	MUC17	608424	Mucin 17, Cell Surface Associated	maintaining homeostasis on mucosal surfaces	/
11p11.2	MYBPC3	600958	the cardiac isoform of myosin-binding protein C	modifies the activity of actin-activated myosin ATPase	63.89%
17p11.2	MYO15A	602666	Unconventional Myosin-15	actin-based motor molecules with ATPase activity	65.06%
10q25.3	NRAP	602873	Nebulin Related Anchoring Protein	anchoring the terminal actin filaments in the myofibril to the membrane and in transmitting tension from the myofibrils to the extracellular matrix	57%
8q21.3	PSKH2	/	Protein Serine Kinase H2	transferase activity and protein tyrosine kinase activity	46%
12q24.13	RITA1	/	RBPJ Interacting and Tubulin Associated 1	Tubulin-binding	/
2q24.3	SCN7A	182392	Sodium Voltage-Gated Channel Alpha Subunit 7	mediates the voltage-dependent sodium ion permeability of excitable membranes	/
12q14.1	SLC16A7	603654	Solute Carrier Family 16 Member 7	symporter activity and secondary active monocarboxylate transmembrane transporter activity	66.93%
7q36.1	SSPO	617356	SCO-Spondin, Pseudogene	modulation of neuronal aggregation	52.3%
5q22.1	TMEM232	/	Transmembrane Protein 232	integral component of membrane	48.42%
11q14.3	TRIM64B	/	Tripartite Motif Containing 64B	metal ion binding	18%
**14q32.12**	**TRIP11**	**604505**	**Thyroid Receptor-interacting Protein 11**	**the maintenance of Golgi structure and function**	**58.42%**
3q25.2	TSEN2	608753	TRNA-Splicing Endonuclease Subunit Sen2	nucleic acid binding and tRNA-intron endonuclease activity.	52.77%

Bold items are candidate genes we identified.

**Table 3:** The bioinformatics information on the LOF variants of candidate genes and patients.

ID	Gene	Mutation site	Amino acid change	Exonic Function	ExAC allele frequency	1000G allele frequency	genomeAD pLoF (pLI)	REVEL score	CADD score
63	*TRIP11*	NM_004239.4:c.5855C>G	p.Ser1952*	stopgain	NA	NA	0	NA	39
24	*TRIP11*	NM_004239.4:c.4432_4433del	p.Glu1478Ilefs*8	frameshift deletion	NA	NA		NA	NA
44	*DNHD1*	NM_144666.3:c.2545C>T	p.Arg849*	stopgain	NA	NA	0	NA	36
36	*DNHD1*	NM_144666.3:c.11206_11207insTT	-	splicing	NA	NA		NA	NA
64	*DNHD1*	NM_144666.3:c.7041dupC	p.Gln2348Profs*21	frameshift insertion	NA	NA		NA	NA
60	*CFAP74*	XM_017002642.1:c.163G>T	p.Glu55*	stopgain	NA	NA	0	NA	28.3
15	*CFAP74*	XM_017002642.1:c.1072del	p.Arg358Glyfs*52	frameshift deletion	NA	NA		NA	NA
3	*CFAP74*	XM_017002642.1:c.1714_1715del	p.Gly572Glnfs*35	frameshift deletion	0.0001	NA		NA	NA
55	*EGR4*	NM_001965.4:c.65dupG	p.Cys22Trpfs*7	frameshift insertion	0.0002	NA	0.01	NA	NA
72	*EGR4*	NM_001965.4:c.65dupG	p.Cys22Trpfs*7	frameshift insertion	0.0002	NA		NA	NA

NA, not available

**Table 4:** Clinical phenotypes of laterality defects patients with LOF variations.

ID	Mutation site	Patients’ cardiac abnormalities	Extracardiac abnormalities
63	NM_004239.4:c.5855C>G	M, ASI, VSS, PA, PDA, ASD, VSD	BI, SRS, RS, LSL
24	NM_004239.4:c.4432_4433del	M, ASI, SV, PS, PDA, ASD	BI, SRS, RS, LSL
44	NM_144666.3:c.2545C>T	D, IRAA, SV, MGA, PS, CAVC	BRB, asplenia, LS, LCS
36	NM_144666.3:c.11206_11207insTT	D, IRAA, SV, MGA, PS, ASD	BRB, SRS, RS, LCS
64	NM_144666.3:c.7041dupC	D, ASI, VSS, TGA, PDA, ASD	BI, SRS, RS, LSL
60	XM_017002642.1:c.163G>T	D, ASI, VSI, TGA, PA, PDA, ASD, VSD	BI, SRS, RS, LSL
15	XM_017002642.1:c.1072del	D, ASI, SV, MGA, PS, PDA, CAVC	BI, SRS, RS, LSL
3	XM_017002642.1:c.1714_1715del	D, IRAA, VSI, DORV, CAVC	BRB, asplenia, LS, RSL
55	NM_001965.4:c.65dupG	D, ILAA, SV, MGA, PA, PDA	BLB, polysplenia, SCS, RSL
72	NM_001965.4:c.65dupG	L, IRAA, SV, MGA, PS, TAPVC, CAVC	BRB, asplenia, RS, LCS

D dextrocardia, M mesocardia, ASI atrial situs inversus, IRAA isomerism of right atrial appendages, ILAA isomerism of left atrial appendages, VSS ventricular situs solitus, VSI ventricular situs inversus, SV single ventricle, PA pulmonary atresia, PS pulmonary stenosis, MGA malposed great arteries, TGA transposed great arteries, DORV double outlet right ventricle, PDA patent ductus arteriosus, CAVC complete atrioventricular canal, ASD atrial septum defect, VSD ventricle septum defect, BI bronchial inversus, BRB bilateral right bronchi (short), BLB bilateral left bronchi (long), SRS single right spleen, SLS single left spleen, RS right-sided stomach, LS left-sided stomach, SCS stomach centrally situated, LSL left-sided liver, RSL right-sided liver, LCS liver centrally situated

To find out whether the patients identified as carriers of the four candidate genes had variants in other known laterality-related genes (*ZIC3*, *CFC1*, *NKX2*.*5*, *GDF1*, *NODAL*, *LEFTY1*, *LEFTY2*, *ACVR2B*, *DNAH5*, *DNAH11*, *DNAI1*, *FOXH1*, *CRELD1*, or *GALNT11*), We screened the coding sequences of these genes. We identified a nonsynonymous heterozygous variant (p.His4123Tyr) in *DNAH5* in one patient with a LOF variant in *CFAP74* (S2 Table).

### Expression patterns of candidate genes in zebrafish

**Fig 2:**
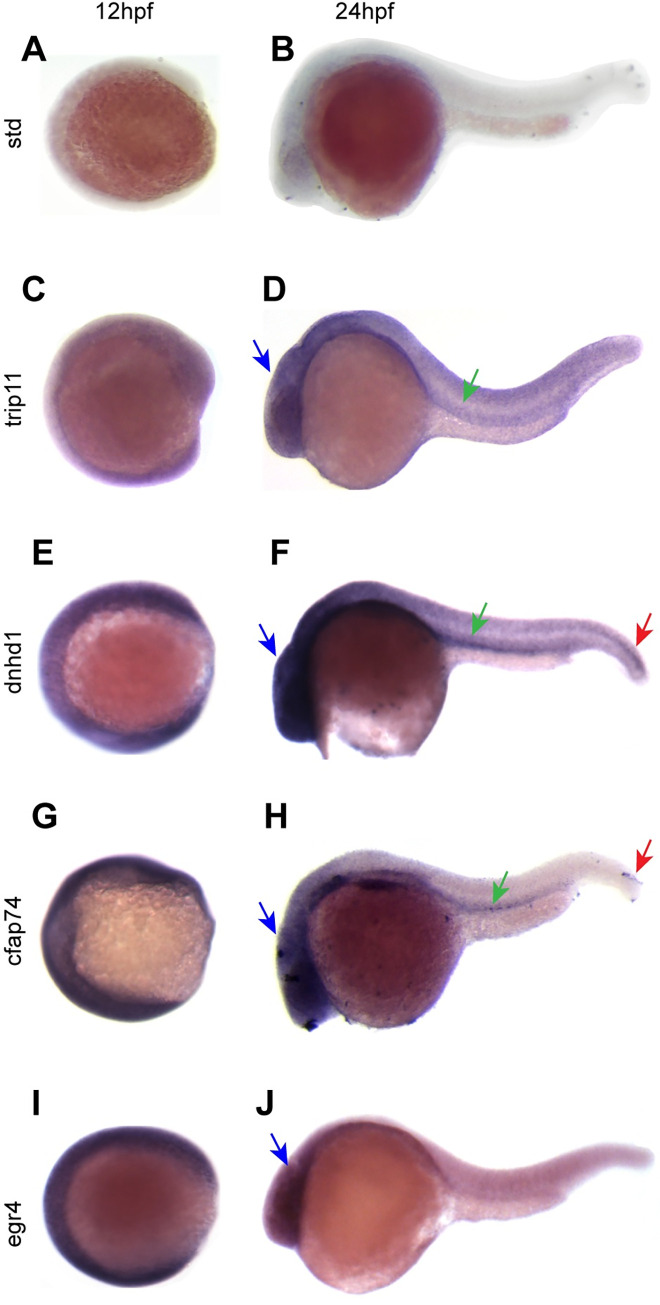
Expression pattern of candidate genes at the indicated stages. 12hpf and 24hpf. (A, C, E, G, I) Results of in situ hybridization of candidate genes and standard control at the 12hpf (8–10 somites). (B, D, F, H, J) Results of in situ hybridization of candidate genes and standard control at the 24hpf (primordium 5 stage). Lateral view of embryos with anterior to the left. pronephric duct (green arrows), tail bud (red arrow), head (blue arrows).

We used zebrafish as a model organism to further analyze the biological function of the candidate genes in regulating organ laterality, as LR patterning processes are highly conserved across vertebrate species. The developmental expression patterns of the candidate genes in zebrafish were examined by whole-mount in situ hybridization at two stages: 12 hpf and 24 hpf. The dorsal forerunner cells cluster and migrate then generate Kupffer’s vesicle (KV) at the tailbud by the 4- to 6-somite stages, which contributes to the proper LR asymmetric patterning in zebrafish [20]. As shown in Fig 2, trip11, dnhd1, cfap74, and egr4 exhibited nearly ubiquitous expression patterns at 12 hpf. Meanwhile, they had more localized expression patterns at 24 hpf: trip11 was localized to the pronephric duct and brain; both cfap74 and dnhd1 were expressed in the pronephric duct, brain, and tailbud; and egr4 mainly expressed in brain. The pronephric duct and neural tube are principal tissues that are involved in ciliogenesis in the early zebrafish embryo [21]. Previous studies in mice showed cilia transduced hedgehog signaling coordinates left-right patterning with heart looping and differentiation of the heart tube [22]. Based on these expression patterns, all candidate genes had a potential role in LR patterning and cardiovascular development.

### Knockdown of candidate genes disturbs cardiac looping

To test whether the candidate genes are required for LR patterning, we used MOs to knock down gene expression by disrupting mRNA splicing (*trip11* and *dnhd1*) or blocking mRNA translation (*cfap74* and *egr4*). We used *galnt11* as a positive control. In previous studies, *GALNT11*, also known as polypeptide N-acetylgalactosaminyltransferase 11, determined laterality by activating Notch signal to regulate the ratio of motile to immotile cilia at the LRO and cilia spatial distribution [23]. Meanwhile, a standard MO provided by Gene Tools was used as a negative control.

**Fig 3:**
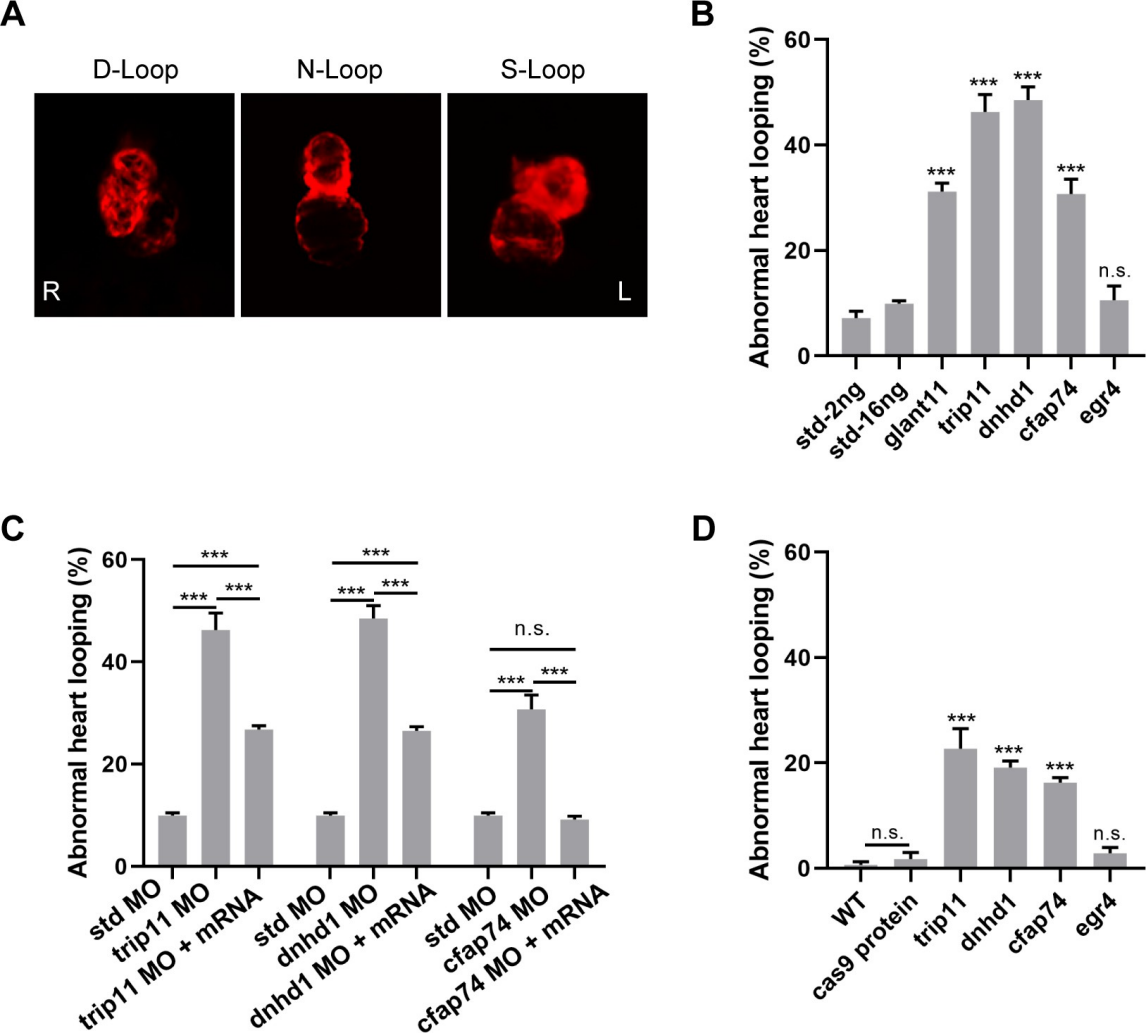
Knock-down of candidate genes disturbed heart looping. (A) Different types of zebrafish heart are shown in Tg (cmlc2: eCherry) morphants in ventral view: dextral loop (normal), sinistral loop (abnormal), and no-loop (abnormal). (B) The percentage of abnormal heart looping with morphants injected. Bars show the total percent of abnormally looped hearts including two types: no-loop and sinistral loop hearts. (C) The corresponding mRNA can rescue LR randomization caused by MO of candidate genes. (D) The ratio of the abnormal heart looping of embryos generated by co-injection of zebrafish Cas9 mRNA and gRNA. Each experiment was repeated at least 3 times with > 50 embryos examined for each group every time. Standard MO (Std) is used as the negative control. galnt11MO is used as the positive control. Chi-squared test (continuity corrected) was used in B, C and D; *P <0.05, **P < 0.01, ***P < 0.001, respectively vs. Std-16ng (*glant11*, *trip11*, *dnhd1*, and *cfap74*) or Std-2ng (*egr4*). WT, wild type.

Three types of heart tube morphologies occur in zebrafish embryos: normal dextral-loop (D-loop), reversed sinistral loop (s-loop), and no loop (Fig 3A). All MOs were injected at the one-cell stage, and embryos were raised until 48 hpf, at which time the cardiac looping morphology was assessed. We found that three of the four candidate gene MOs exhibited a robust cardiac looping phenotype. 7.2–9.8% of the negative controls exhibited an abnormal phenotype, while 31.2% of *galnt11* morphants exhibited an abnormal phenotype (*P* < 0.001). The phenotypes of the *trip11*, *dnhd1*, *and cfap74* morphants were significantly different from that of the negative control, with 30.7–48.5% of embryos showing either an S-loop or no loop (*P* < 0.001). Yet knockdown of *egr4* had no significant effect on heart looping (P > 0.05) (Fig 3B). In addition, when Cas9 protein and guide RNA (gRNA; *trip11* gRNA, *dnhd1* gRNA, and *cfap74* gRNA) were co-injected into one-cell stage zebrafish embryos, both injected and F0 embryos exhibited a disrupted cardiac looping ratio (*P* < 0.001) compared to the negative control (injected with cas9 protein only; Fig 3D). However, knockout of *egr4* had no significant effect on heart looping compared with the negative control. In detail, 0–3.9% of negative controls exhibited an abnormal phenotype, while the percentages of *trip11*, *dnhd1*, *cfap74*, and *egr4* exhibiting abnormal phenotypes were 18.99–26.56%, 18.06–20.51%, 15–17.3%, and 1.59–3.75% respectively.

We then performed rescue experiments to verify whether the knockdown of each candidate gene was responsible for the abnormal phenotype. The defective LR asymmetry of the heart in the *trip11*, *dnhd1*, and *cfap74* morphants were efficiently rescued by expression of the corresponding mRNAs of the candidate genes in vitro and the detailed abnormal ratio after rescue ranged from 9.18% to 26.75% (Fig 3C; *P* < 0.05). Among them, there was no significant difference between *cfap74* morphants and std MO after rescue.

### Candidate genes exhibit global effects on early signaling pathways in LR development

Disrupted cardiac looping patterns can arise either by disturbances during early embryonic LR pattern development or by later morphogenesis of specific internal organs.[24] Clinical data from patients with selected variants indicated more than one organ malposition, suggesting that abnormal cardiac looping results from a disruption of early LR pattern development. To pinpoint the molecular cause of the LR defects, we examined the expression patterns of spaw, lefty2, and pitx2 in the morphants. These three genes are markers of LR patterning. In zebrafish embryos, Kupffer’s vesicle initiates asymmetric orientation by inducing the lateral expression of spaw in the LPM. Then, spaw stimulates the transcription of the downstream genes *lefty2* and *pitx2*, particularly in the left side of the LPM and heart.[25] *Lefty2* encodes a nodal inhibitor belonging to the TGF-β superfamily, whereas *pitx2* encodes a transcription factor that transfers LR patterning information necessary for proper organogenesis [26].

**Fig 4:**
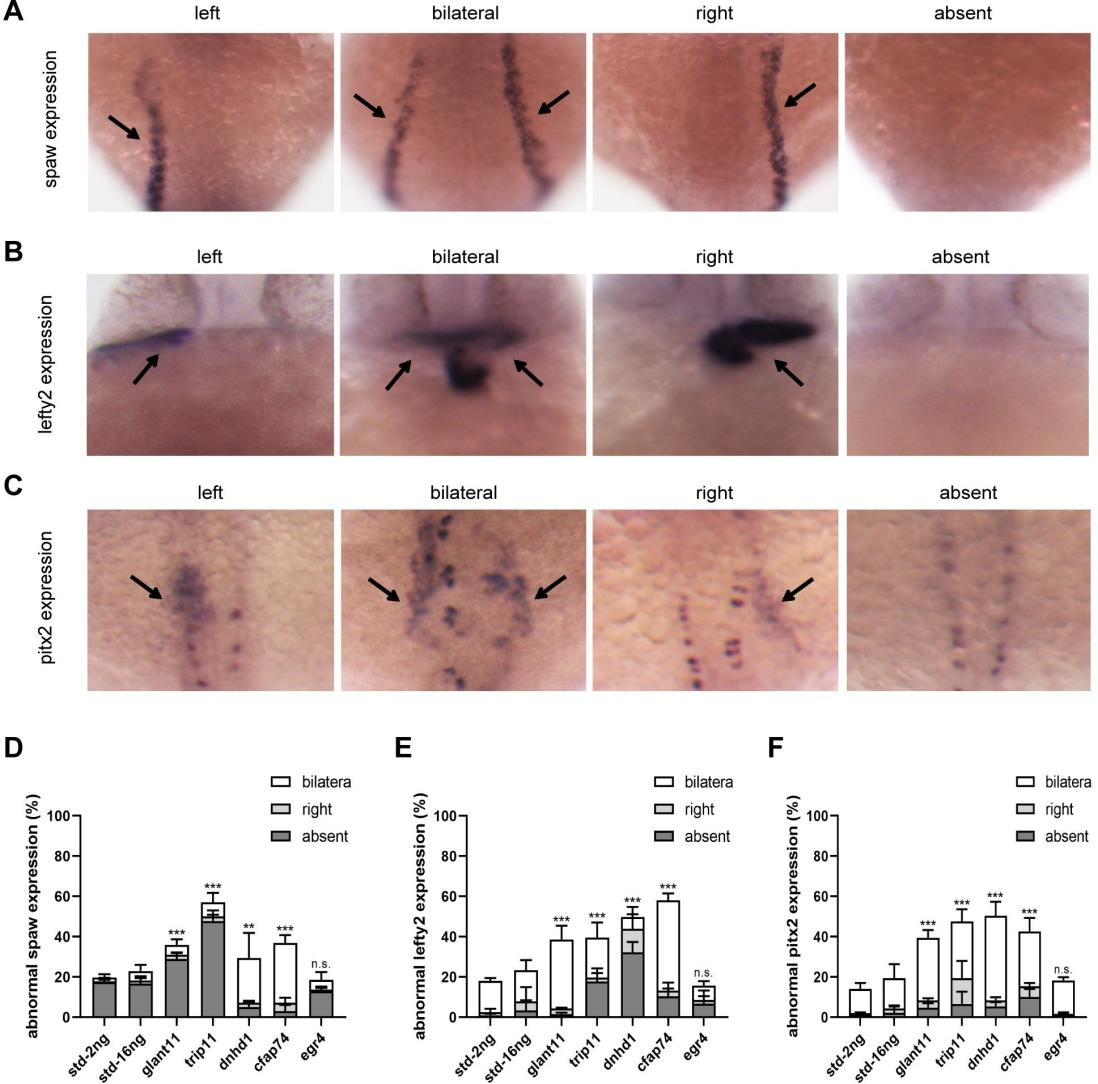
The expression patterns of *spaw*, *lefty2*, and *pitx2* at 18–24 hpf. (A, C) the expression of *spaw* and *pitx2* exhibit four patterns in LPM: left (normal), right (abnormal), bilateral (abnormal), or absent (abnormal). (B) The expression of *lefty2* shows four patterns in the cardiac field: left (normal), right (abnormal), bilateral (abnormal), or absent (abnormal). (D-F) Summary of *spaw*, *lefty2*, and *pitx2* mRNA expression in zebrafish morphants. Embryos are viewed dorsally with anterior to the top. Each experiment was repeated at least 3 times with > 50 embryos examined for each group every time. Chi-squared test (continuity corrected) was used in D, E and F; *P <0.05, **P < 0.01, ***P < 0.001, respectively vs. Std-16ng (*glant11*, *trip11*, *dnhd1*, and *cfap74*) or Std-2ng (*egr4*).

The embryonic expression patterns of spaw, lefty2, and pitx2 exhibited either normal (left side) or abnormal (right side, bilateral, or absent) forms (Fig 4A–4C). Of the negative controls, 14.0–19.4% exhibited abnormal pitx2 expression, 18.1–23.4% displayed abnormal lefty2 expression, and 19.8–22.9% exhibited abnormal spaw expression. Meanwhile, in morphants injected with *galnt11* MO as a positive control, 39.5% exhibited abnormal pitx2 expression, 38.6% displayed abnormal lefty2 expression, and 36.0% exhibited abnormal spaw expression (*P* < 0.001). The *trip11*, *dnhd1*, and *cfap74* morphants exhibited significant abnormal spaw, lefty2, and pitx2 expression patterns (42.6–50.4% of pitx2, 39.7–58.1% of lefty2, and 29.4–57.1% of spaw; *P* < 0.01) compared with negative control. Consistent with the phenotypic results, *egr4* morphants exhibited no significant abnormalities in spaw, lefty2, or pitx2 expression. (Fig 4D–4F)

### The role of candidate genes in Kupffer’s vesicle organogenesis and ciliogenesis

**Fig 5:**
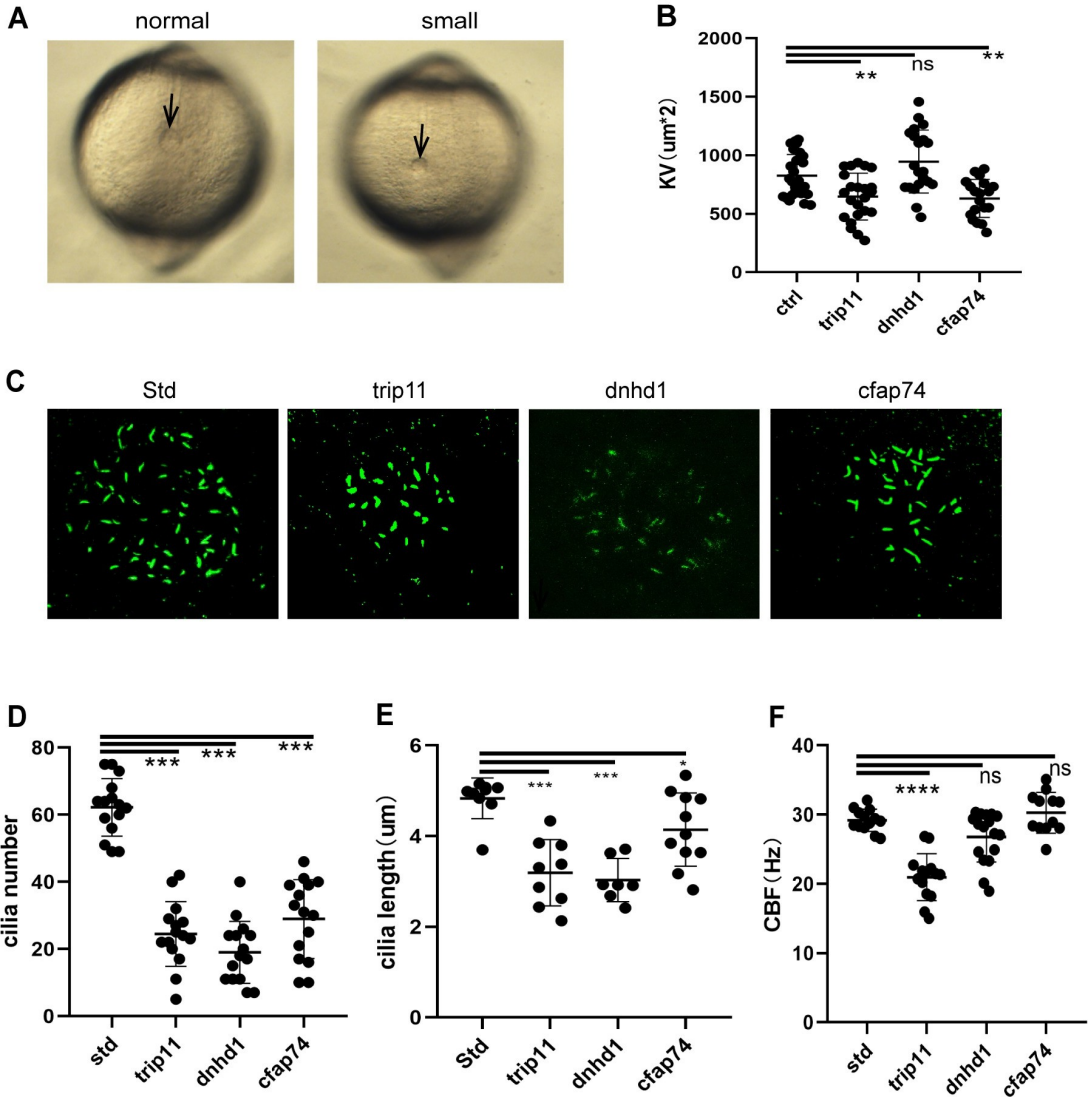
Candidate genes are required for KV formation and ciliogenesis. (A) The light-micro graph at the 8-somite stage showed different KV sizes, including normal and small. (B) The vesicle size of KV apical area. N≥19. (C) Fluorescent immunostaining of cilia in KV using anti-α-Tubulin antibodies at the 12hpf. (D) LRO Cilia number per KV was quantified and the group values were expressed as the mean ± SD. N≥15. (E) The average length of LRO Cilia per KV was quantified and the group values were expressed as the mean ± SD. N≥7. (F) The ciliary beat frequency (CBF). N≥12. Statistical significance was determined by Mann Whitney test; *P <0.05, ***P < 0.001, respectively vs. Std.

Kupffer’s vesicle (KV) is a conserved ciliated epithelial structure that creates nodal flow by the directional rotation of the cilia. This flow is necessary for asymmetric gene expression [1]. Our results revealed that *trip11*, *dnhd1*, and *cfap74* might act upstream of *spaw*, impacting Nodal signaling in early development. To investigate whether the loss of these candidate genes alters LR asymmetric gene expression KV organogenesis or ciliogenesis, we first examined the morphogenesis of KV. Compared with wild-type embryos exhibiting a normal-size, rounded KV at the terminus of the notochord, *trip11*, and *cfap74* morphants displayed a smaller KV. (Fig 5A and 5B)

To explore the function of the candidate genes during ciliogenesis, the formation of KV cilia in zebrafish embryos was analyzed at the 8-somite stage. Compared with the number (average, 62±9) and length (average, 4.83±0.42 μm) of cilia in control embryos, *trip11* morphants exhibited a significant decrease in the mean number (24±10) and mean length (3.19±0.69 μm) of cilia (*P* < 0.001). Consistently, *dnhd1* morphants showed similar abnormalities in ciliogenesis, with an average number of 19±9 and an average length of 3.03±0.44 μm (*P* < 0.001); the average number of cilia in *cfap74* embryonic morphants was 29±12 (*P* < 0.001) and the average length was 4.14±0.77 μm (*P* < 0.05; Fig 5C–5E). Moreover, we injected arl13b-mCherry mRNA into KVs at the one-cell stage to track the movements of the cilia at the 8-somite stage. The ciliary beat frequency (CBF) in the KV of trip11 knockdown embryos was reduced significantly (Fig 5F). Dnhd1 and cfap74 knockdown embryos showed no significant difference compared with control embryos (**S1–S4 Movies**).

### Variant screening of candidate genes

We re-screened 70 patients with laterality defects for rare nonsynonymous variants of three candidate genes (*TRIP11*, *DNHD1*, and *CFAP74*) to further investigate the relationship between these genes and LR patterning. We then screened the sequences using the following criteria: (1) located in exon or splicing region; (2) exclude synonymous variants; (3) exclude variants with allele frequency >0.1% in 1000 Genomes Project or ExAC; (4) exclude variants identified in 100 normal Chinese individuals; (5) predicted to be disease-causing by at least one online program. Finally, ten rare heterozygous variants in *DNHD1* and *CFAP74* were identified S3 Table. The clinical phenotypes of these patients were shown in S4 Table.

## Discussion

Laterality defects can lead to a variety of congenital diseases, but the etiology of these defects in many patients is still unknown [27]. To explore the role of genetic variation in laterality defects, we performed ES on 70 unrelated patients and 100 healthy individuals. By analyzing the sequencing results, we identified four candidate genes. We assayed for phenocopy in a zebrafish model and then embarked on mechanistic analyses to understand the roles of these candidate genes in LR patterning and related diseases. The downregulation of these genes (*trip11*, *dnhd1*, and *cfap74*) in zebrafish resulted in disorders of both cardiac looping and the ectopic expression of nodal-responsive genes (*spaw*, *lefty2*, and *pitx2*). Meanwhile, knockdown of *trip11*, *dnhd1*, and *cfap74* altered the formation and function of cilia in KV. In addition, we identified 10 rare nonsynonymous variants in the coding sequences of *DNHD1* and *CFAP74* in patients with laterality defects.

Currently, more than 100 genes have been associated with LR patterning defects in animal models, but only a few are likely candidates in humans [1]. Many studies have shown that using ES is an efficient strategy to identify pathologic variations of genes related to disease. However, since the acquisition of the patient population in this study was random, and the high number of variants identified would likely interfere with our analysis, it was critical to optimize the screening criteria for the identification of candidate genes in humans. Among all genetic variants, LOF variants generally exhibit strong pathogenicity [28]. The genetic basis of disorders such as congenital isolated hypogonadotropic hypogonadism, Aicardi-Goutiéres syndrome, and cavernous angioma have demonstrated LOF variants that are of considerable importance in etiological research [29–31]. Moreover, previous studies of *NEK3*, *MMP21*, and *PKD1L1* have revealed that LOF variants play important roles in the pathogenesis of laterality defects in humans [3,32,33]. Thus, we screened 70 unrelated patients for rare, LOF variants that were present more than once in the same gene to narrow the scope of candidates. According to our zebrafish models, three out of four potential genes were shown to be involved in the development of asymmetry. These findings confirm that focusing on rare, LOF variants is a good approach to identify candidate genes, and the presence of more than one LOF variant in the same gene is more likely to elicit abnormal functional effects.

Our results suggest that *TRIP11*, *DNHD1*, and *CFAP74* are involved in Nodal signaling cascade by participating in KV development or ciliogenesis, which in turn contributes to LR pattern formation. These results confirm the importance of KV in LR patterning. Established genes of left–right patterning defects often involve the left-right organizer (LRO) [34]. According to the research conducted by Jason et al, the size of the KV lumen may have a significant impact on the flow dynamics necessary for LR development [35]. Besides, these results indicate the importance of ciliogenesis and ciliary function in left-right patterning. A previous study performed by Knowles et al showed that around 50% of primary ciliary dyskinesia patients have organ laterality defects [36].

The thyroid hormone receptor interactor 11 (*TRIP11*) gene encodes the Golgi microtubule-associated protein 210 (GMAP-210), which belongs to the golgin family and has been proposed to function in maintaining the morphologic and function of the Golgi apparatus [37]. Former research has shown that the loss of *TRIP11* results in developmental problems related to the defective formation and function of cilia. The lack of GMAP-210 in mice has been shown to cause lethality in neonates with diverse phenotypes, including growth restriction, tetralogy of Fallot, ventricular septal defects, and lung hypoplasia [38]. In addition, *TRIP11* male germ cell-specific conditional knockout mice exhibit infertility [39]. Heart and lung development, as well as male fertility, require normal ciliary function. Furthermore, cilia are of great importance in LR asymmetry. Our study is the first to show that *trip11* exhibited ubiquitous expression patterns at 12 hpf and localized to the pronephric duct and brain at 24 hpf in zebrafish embryos. *Trip11* knockdown led to apparent abnormalities in cardiac looping. Further, cultured mouse embryonic kidney cells obtained from GMAP-210-deficient mice displayed shortened cilia with reduced polycystin-2 levels in a previous study [38]. Interestingly, we observed similar length defects in *trip11* morphants and found that *trip11* knockdown impaired ciliary motion. It is known that GMAP-210 anchors IFT20 to the Golgi apparatus, which is required for IFT20 to sort and transport ciliary membrane proteins, such as polycystin-2 [37]. Previous work has indicated that polycystin-2 acts as a regulator of cilia length to participate in the regulation of flow-induced signaling. Polycystin-2 is also required for sensing ciliary motility in LR axis determination [40]. The involvement of both IFT20 and polycystin-2 may explain why impairments in both the structure and function of cilia were observed in *trip11* morphants.

Few studies have been performed on the function of either *DNHD1* or *CFAP74*. *DNHD1*, dynein heavy chain domain 1, encodes a ciliated structural protein in the dynein heavy chain. A homozygous missense variant of *DNHD1* was reported in patients with SI-like phenotypes (complex heart defects with incomplete intestinal rotation), but no further studies were conducted. Noteworthy, research conducted by Yue-Qiu Tan showed that *DNHD1* bi-allelic variants were identified in athenoteratozoospermia patients and these patients presented flagellar axoneme defects [41]. DNHD1 may be involved in the developmental process by participating in cilia assembly. Previous research has noted the association of several other dynein heavy chain-encoding genes, such as *DNAH1*, *DNAH5*, *DNAH9*, and *DNAH11*, with ciliary primary dyskinesia (PCD) and laterality defects [42–45]. PCD is a serious inherited disorder that results from defects in ciliary and flagellar axoneme substructures with LR laterality developing in 50% of affected individuals [7]. Among these genes, a deficiency in *Dnah5*, a paralog of *Dnhd1*, causes a loss of outer dynein arms (ODAs) in embryonic LRO monocilia, leading to immotile cilia and impaired fluid flow in mice [13]. However, unlike *Dnah5*, loss of *dnhd1* did not elicit any impairment in ciliary motion, so we considered the loss of cilia number and length as influential factors of nodal flow. We first found that the loss of dnah1 causes disturbances in cardiac looping and exhibited global defects in early signaling pathways in zebrafish, which likely result from impaired ciliogenesis.

*CFAP74* (cilia- and flagella-associated protein 74) is a protein-coding gene that is reportedly linked to olfactory function [46]. It is highly expressed in the testes and lungs, with normal cilia playing crucial roles in both sperm motility and respiratory function.[47] Recently, biallelic mutations in *CFAP74* were identified in two patients with potential PCD and multiple morphological abnormalities of sperm flagella (MMAF), but no laterality defects were found in these patients [48]. Abnormalities in CFAP family members appear to be associated with both PCD and MMAF. For example, *CFAP53* was found in patients with laterality disorders; its deficiency in animal models was shown to result in anomalous LR asymmetry [49–51]. Further, the specific knockdown of *CFAP53* in zebrafish exhibited ultrastructural defects characterized by the severe reduction of ODAs and nonmotile cilia in KV [50,51]. Prediction of CFAP74 function based on its protein structure suggests a role in the ciliary movement, as it is part of the central apparatus of the cilia axoneme. Previous research provided indirect evidence of this function by showing that a mutation in *CFAP74* interfered with the assembly of the axoneme structure and function of the sperm flagellum [48]. Therefore, *CFAP74* loss of function was considered to disrupt the structure of cilia with a reduction in both the number and length of cilia. Similar to *DNHD1*, the loss of *CFAP74* caused the loss of cilia number and length, while did not impair ciliary motion, so we considered loss of *CFAP74* influencing ciliary function by affecting its formation. Based on these results, we suspect that *TRIP11*, *DNHD1*, and *CFAP74*, which are structurally related ciliary proteins in KV, act upstream of the Nodal signaling cascade and their decreased expression affects ciliary function.

As previously described, a single genetic mutation may not result in an obvious phenotype, but mutations in highly pleiotropic genes could have subclinical effects on phenotypes, and their cumulative effect could impact the fitness of its carrier [52]. Hence, we took the additive effects of different variants into account in our study; such considerations have also been proposed in autism spectrum disorder, mitochondrial encephalomyopathy, and several cancers [53–55]. Similar effects may exist in patients with LOF variants in *CFAP74* who also have one nonsynonymous heterozygous variant (p.His4123Tyr) in *DNAH5*. The multi-genetic basis of our results is consistent with the complexity of the development of LR asymmetry; however, to verify this hypothesis, experimental modeling and further research are needed.

Early growth response 4 (*EGR4*) is a transcriptional regulator involved in mitogenesis and differentiation [56]. In our study, knockdown of *EGR4* in zebrafish did not exhibit abnormal LR patterning.

Our research highlights the importance of rare, LOF variants in the identification of novel candidate genes in random patients with laterality defects. In addition, our functional studies illustrate those three potential genes, two of which have never been associated with LR asymmetry in either humans or animals previously, may be essential for the development of LR patterning. However, according to the guidelines, we do not have sufficient experimental and informatic support that the variants in the three genes are causal for laterality defects [57,58]. This is the limitation of our study. *TRIP11*, *DNHD1*, and *CFAP74* are more likely to be candidates with new roles in left-right patterning. Meanwhile, *TRIP11* and *CFAP74* are associated with laterality defects for the first time. We provide preliminary evidence for their potential pathogenicity in laterality defects, and they are hoped to be further confirmed in larger groups of patients in the future. Overall, these findings have broadened our insights into the complex genetics of laterality defects and the pathogenic mechanism involved.

## Methods

### Ethics statement

This study was approved by the Local Ethics Committees of Xinhua Hospital (Shanghai, China) and Shanghai Children’s Medical Center (SCMC). The serial numbers are XHEC-C-2012-018 and SCMC-201004 respectively. All parents have signed informed consent according to the guidelines of the medical ethics committee of Xin Hua Hospital and SCMC.

### Case ascertainment

Our study recruited 70 patients with laterality defects and 100 healthy individuals from Xinhua Hospital and Shanghai Children’s Medical Center (SCMC). All patients included in this research were diagnosed with laterality defects and confirmed by ultrasonography, echocardiography, cardiac catheterization examinations, X-ray, computed tomography, and other operation recordings.

Patients exhibiting complex congenital heart defects and abnormal arrangement of the visceral organs were included, while those with a known chromosome abnormality or Mendelian gene syndrome and another major congenital malformation not associated with laterality defects were excluded.

### Exome sequencing and variants screening

DNA was extracted from peripheral blood samples obtained from each patient using DNeasy Blood Kit (Qiagen, Duesseldorf, Germany) according to the manufacturer’s instructions. DNA of the cases and controls were sent to a commercial provider (Shanghai Biotechnology Co., Ltd., Shanghai, China), which performed sequencing services using the Illumina HiSeq2500 platform. Read mapping to hg19 was performed with Burrows-Wheeler alignment (BWA (0.7.12)). The coverage is more than 99% with a mean depth of more than 60x. The variants were annotated by ANNOVAR with a combination of databases.

Variants with more than 0.001 alternative allele frequencies in 1000 Genomes Project (http://www.1000genomes.org/) and ExAC (http://exac.broadinstitute.org), as well as those existing in control individuals were excluded. Pathogenicity of all the variants was predicted by online programs including SIFT (http://provean.jcvi.org/index.php), Polyphen-2 (http://genetics.bwh.havard.edu/pph2/), Mutation Taster (http://www.mutationtaster.org/), gnomeAD (http://gnomad-sg.org/about), REVEL (https://sites.google.com/site/revelgenomics/) and CADD (http://cadd.gs.washington.edu/score). The variants identified were confirmed by Sanger sequencing. The amplification reactions were carried out on an Applied Biosystems Veriti Cycler (Life Technologies Corporation, USA) with the following cycling program: 98°C for 5 minutes and amplified for 35 cycles, each consisting of 30 seconds at 98°C, 30 seconds at 55–63°C, and 30 seconds at 72°C per 1kb, followed by a 3-minute extension at 72°C. The sequences of Sanger sequencing primers are provided in S5 Table.

### Zebrafish strains

Adult zebrafish (albino and AB line) and red-fluorescent labeled zebrafish (cmcl2: mcherry) were raised under standard laboratory conditions using an automatic fish housing system (ESEN, Beijing, China) at 28°C. Wild-type embryos were obtained from adult zebrafish and raised in Holtfreter’s solution at 28.5°C. All zebrafish experiments were performed at the Institute of Neuroscience, Chinese Academy of Sciences, under the guidelines of standard protocols. The stages of embryos were determined according to their developmental morphology [59].

### The whole-mount in situ hybridization

The whole-mount in situ hybridization was performed according to the previously described protocol [60]. Probes of the following genes were used in our research: *spaw*, *pitx2*, *lefty2*, *trip11*, *dnhd1*, *cfap74*, *egr4*. The anti-DIG RNA probes were synthesized with a length of 600–1300 nucleotides. Among them, the probes of *trip11*, *dnhd1*, *cfap74*, and *egr4* were synthesized by the company GENEWIZ (Suzhou, China) and then subcloned into the pGEM-T vector. The coding sequence DNA of *spaw*, *pitx2*, and *lefty2* was amplified using specific primers and then also subcloned into the pGEM-T vector. The sequences and primers are listed in S6 Table.

### Morpholino oligo injection and target gene knockdown

The standard control morpholino oligo (MO) and MOs targeting candidate genes were purchased from Gene Tools (Philomath, OR, USA). The standard control MO is a 25-mer oligo with the sequence: 5’-CCTCTTACCTCAGTTACAATTTATA-3’. According to Gene Tools’ protocol, MOs were diluted to different working concentrations using nuclease-free water and were pressure-injected into one-cell-stage embryos by a Picospritzer II injector. The MOs for examining heart looping and scoring of *pitx2*, *lefty2*, and *spaw* expression in morphants were used in dosages ranging from 2 ng to 16 ng: 4ng *trip11* MO, 4ng *dnhd1* MO, 16ng *cfap74* MO, 2ng *erg4* MO, and 8ng *galnt11* MO per embryo. As negative controls, we injected 2 and 16 ng of standard control MO separately. A summary of MO doses and sequences is provided in S7 Table.

### The effectiveness evaluation of the MOs

The effectiveness evaluation methods of candidate genes are different based on different MO principles. The effectiveness evaluation method of MOs that inhibit splicing (splicing MO) targeting *trip11*/*dnhd1* is to directly detect whether the normal splicing of the original mRNA transcript has been changed by RT-PCR. The RT-PCR validation was performed according to the protocol of SYBR Premix Ex Taq II (Applied TaKaRa, Japan). The sequences of three pairs of primers are provided in S8 Table.

The MOs targeting *cfap74* and *egr4* are translation-inhibiting MO (MO-ATG). We measured the effectiveness of *egr4* MO by western blotting, while determining that of *cfap74* MO by in vitro reporter gene methods as lacking zebrafish antibodies against *cfap74*. Briefly, a pair of oligos that contain the MO target sequence of candidate genes were first annealed and then recombined into PeGFP-N1 vector which expressed the fused construct consisting of MO target sequence and the coding sequence of eGFP. 100 pg of the fusion gene vector and 16ng of the *cfap74* MO or control MO were microinjected into each zebrafish embryo at the one-cell stage. The gene which expressed fluorescent protein eGFP was observed under the fluorescence microscope. The knockdown efficiencies of these MOs are illustrated in S3 Fig.

### CRISPR/Cas9-mediated gene editing

The sequences of guide RNA were designed to introduce *trip11*, *dnhd1*, and *cfap74* gene mutation in zebrafish embryos by the clustered regularly interspaced short palindromic repeats (CRISPR)/CRISPR-associated protein 9 (Cas9) system [61]. The sequences of *trip11*, *dnhd1*, *cfap74*, and *egr4* guide RNA (gRNA) were designed to target the sequences of mature genes and constructed by the manufacturer (XINJIA Medical, Nanjing, China). According to the previously described protocol, 600 pg zCas9 protein, and 100 pg-250 pg candidate genes, gRNA was co-injected into zebrafish embryos at the one-cell stage. The specific sequences of gRNA of candidate genes are listed in S9 Table. Then, we examined the knockout efficiency in F0 embryos by PCR and sequencing analysis. The sequences of primers are listed in S10 Table and the results are shown in S4 Fig. The knockout efficiency of *trip11*, *dnhd1*, *cfap74*, and *egr4* is 100%, 62.5%, 100%, and 100%, separately.

### mRNA synthesis and injection

The full-length coding sequence DNA of *trip11*, *dnhd1*, *cfap74*, and *egr4* were synthesized by the company GENEWIZ (Suzhou, China) and then subcloned into the pCS2+ vector. The positive clones were selected by DNA sequencing to be applied for generating full-length mRNAs. The corresponding mRNAs of candidate genes were generated by T7 or SP6 mMessage mMachine kit (Ambion, America). For the rescue experiment, the mRNA and MO of candidate genes were mixed and injected into one-cell-stage embryos. The dose of mRNAs and MOs are listed in S11 Table.

### Immunostaining and confocal microscope

Embryos were fixed in 4% paraformaldehyde in PBS overnight at 4°C, followed by dehydration in 100% ethanol at 20°C. Embryos were rehydrated by moving into successive dilutions of methanol in PBS and then rinsed with PBST two times every 5 minutes. Embryos were then blocked at room temperature for 2 hours in 10% heat-inactivated goat serum and then stained with the anti-α-Tubulin antibody (1:2000 T7451, Sigma) overnight at 4°C. Samples were then washed 3 times with PBST, followed by incubation with secondary antibodies, Alexa Fluor 488 conjugated anti-mouse IgG (1:500 115-545-003, Invitrogen), overnight at 4°C. The stained embryos were then embedded with 1.5% low melting agarose and imaged using an Olympus FV3000 confocal. We measured the cilia length by 3D tracing of cilia with imageJ. The number of KV and cilia counted were shown in S12 Table.

KV apical area was quantified to visualize the KV. Embryos were observed in a bright field using an Olympus SZX7 microscope at the 8-somite stage. A region was drawn around the KV apical perimeter and then measured using ImageJ software (NIH) to quantify the vesicle size.

### High-speed cilia video microscopy

Embryos at the eight-somite stage were mounted in 1.2% agarose with the dorsal roof of the KV facing up. Movie capture was performed at 125–250 frames per second under the OLYMPUS XLPLN25XSVMP2 25x/1.00 WD 4.00 mm objective lens on a Bruker Opterra II controlled with Prairie View Software at room temperature. CBF measurements were analyzed using ImageJ (NIH) followed by Fourier analysis in MATLAB as previously described [62].

### Statistical analysis

Cilia number and length were measured using ImageJ software. All results were expressed as the mean ± SD. Differences between control and treated groups were analyzed using the chi-squared test (continuity corrected), unpaired, two-tailed t-test, and Mann-Whitney test. Results were collected from at least 3 biologically independent replicates and considered statistically significant at P < 0.05 and defined *P <0.05, **P < 0.01, ***P < 0.001.

## Supporting information

S1 Fig Sanger sequencing shows loss-of-function variants. (a, b) Sanger sequencing shows frameshift or nonsense variants in *TRIP11*. (c, g, h) Sanger sequencing shows frameshift, splice-region mutant alleles or nonsense variants in *DNHD1*. (i, m, n) Sanger sequencing shows frameshift or nonsense variants in *CFAP74*. (o, s) Sanger sequencing shows frameshift variants in *EGR4*. (d, e, f, j, k, l, p, q, r, v) Sanger sequencing shows normal results that did not alter the sequences.
(TIF)

S2 Fig Schematic representation of the domains of four candidate genes and the position variants. (a) the position of domains and variants in *TRIP11*. ALPS, ALPS (amphipathic lipid-packing sensor) motif; GRAB, GRAB (Grip-related Arf-binding) domain; GA1, GRAB-associated region. (b) the position of domains and variants in *DNHD1*. MTBD, microtubule-binding domain. (c) the position of domains and variants in *CFAP74*. TPH, Trichohyalin-plectin-homology domain. (d) the position of domains and variants in *EGR4*.
(TIF)

S3 Fig The effectiveness of the MOs. (a-b) The RT-PCR (reverse transcription-PCR) results were conducted to analyze the efficiency of sb-MOs targeting *trip11*, and *dnhd1*. Total RNA was extracted from 2 dpf zebrafish embryos. (a) The *trip11* splice blocking morpholino (sb-MO) targets the junction of intron 1–2 and exon 2 resulting in a shorter exon 2. (b) The *dnhd1* sb-MO target the junction of exon 2, and intron 2–3 results in a shorter exon 2. (c,d) The *egr4* MO and *cfap74* MO target AUG result in lower protein expression. (c) Fluorescent immunostaining of zebrafish embryo using anti-GFP antibodies in Std embryos and *cfap74* morphants. The fusion gene vector and *cfap74* MO or control MO were microinjected at the one-cell stage. (d) Western blot revealed knockdown of protein expression in *egr4* morphants. Anti-actin was used as a loading control. Proteins were extracted from 3 dpf zebrafish embryos. Std standard control; ex, exon; in, intron.
(TIF)

S4 Fig Sequencing analysis of the knockout results. Sequence analysis of *trip11*, *dnhd1*, *cfap74*, and *egr4* mutations caused by co-injection of zebrafish codon-optimized protein and corresponding gRNA. The red fonts show the target sites of gRNA, yellow fonts and blanks show mutated sequences, and the green fonts show the PAM sequences.
(TIF)

S1 Table The bioinformatics information of 776 candidate LOF variants. (XLSX)

S2 Table The bioinformatics information on the variants of patients with selected LOF mutations. (DOCX)

S3 Table The bioinformatics information on the variants of candidate genes. (DOCX)

S4 Table Clinical phenotypes of laterality defects patients with rare nonsynonymous variants of three candidate genes. (DOCX)

S5 Table The primers of Sanger sequencing. (DOCX)

S6 Table Antisense RNA probes conducted for whole mount in situs hybridization. (DOCX)

S7 Table MO sequences, injection doses, and total embryo numbers analyzed for heart looping and gene expression. (DOCX)

S8 Table The primers of MOs’ effectiveness evaluation. (DOCX)

S9 Table The sequences and doses of gRNA. (DOCX)

S10 Table The primers of CRISPR/Cas9-mediated gene editing effectiveness evaluation. (DOCX)

S11 Table MO and mRNA injection doses, and total embryo numbers analyzed for rescue. (DOCX)

S12 Table Total number of KV and cilia for cilia length and CBF. (DOCX)

S13 Table The numerical data that underlie the figure and statistics. (XLSX)

S1 Movie The motion of motile cilia in control morphants. (RAR)

S2 Movie The motion of motile cilia in trip11 morphants. (RAR)

S3 Movie The motion of motile cilia in dnhd1 morphants. (RAR)

S4 Movie The motion of motile cilia in cfap74 morphants. (RAR)

## References

[1] Catana A, Apostu AP: The determination factors of left-right asymmetry disorders- a short review. Clujul Med. 2017; 90:139–146. doi: 10.15386/cjmed-701 28559696 PMC5433564

[2] Shiraishi I: Left-Right Asymmetry and Human Heterotaxy Syndrome. In Etiology and Morphogenesis of Congenital Heart Disease: From Gene Function and Cellular Interaction to Morphology. Edited by Nakanishi T, Markwald RR, Baldwin HS, Keller BB, Srivastava D, Yamagishi H. Tokyo: Springer Copyright. 2016; 2016: 49–56 29787042

[3] Guimier A, Gabriel GC, Bajolle F, Tsang M, Liu H, Noll A, Schwartz M, El Malti R, Smith LD, Klena NT, et al: MMP21 is mutated in human heterotaxy and is required for normal left-right asymmetry in vertebrates. Nat Genet. 2015; 47:1260–1263. doi: 10.1038/ng.3376 26437028 PMC5620017

[4] Escobar-Diaz MC, Friedman K, Salem Y, Marx GR, Kalish BT, Lafranchi T, Rathod RH, Emani S, Geva T, Tworetzky W: Perinatal and infant outcomes of prenatal diagnosis of heterotaxy syndrome (asplenia and polysplenia). Am J Cardiol. 2014; 114:612–617. doi: 10.1016/j.amjcard.2014.05.042 24996551 PMC4307386

[5] Nakhleh N, Francis R, Giese RA, Tian X, Li Y, Zariwala MA, Yagi H, Khalifa O, Kureshi S, Chatterjee B, et al: High prevalence of respiratory ciliary dysfunction in congenital heart disease patients with heterotaxy. Circulation. 2012; 125(18):2232–2242. doi: 10.1161/CIRCULATIONAHA.111.079780 22499950 PMC3770728

[6] Gabriel GC, Lo CW: Left-right patterning in congenital heart disease beyond heterotaxy. Am J Med Genet C Semin Med Genet. 2020; 184:90–96. doi: 10.1002/ajmg.c.31768 31999049 PMC7261368

[7] Wallmeier J, Nielsen KG, Kuehni CE, Lucas JS, Leigh MW, Zariwala MA, Omran H: Motile ciliopathies. Nat Rev Dis Primers. 2020; 6:77. doi: 10.1038/s41572-020-0209-6 32943623

[8] Loges NT, Antony D, Maver A, Deardorff MA, Güleç EY, Gezdirici A, Nöthe-Menchen T, Höben IM, Jelten L, Frank D, et al: Recessive DNAH9 Loss-of-Function Mutations Cause Laterality Defects and Subtle Respiratory Ciliary-Beating Defects. Am J Hum Genet. 2018; 103:995–1008. doi: 10.1016/j.ajhg.2018.10.020 30471718 PMC6288205

[9] Bamford RN, Roessler E, Burdine RD, Saplakoğlu U, dela Cruz J, Splitt M, Goodship JA, Towbin J, Bowers P, Ferrero GB, et al: Loss-of-function mutations in the EGF-CFC gene CFC1 are associated with human left-right laterality defects. Nat Genet. 2000; 26:365–369. doi: 10.1038/81695 11062482

[10] Sutherland MJ, Wang S, Quinn ME, Haaning A, Ware SM: Zic3 is required in the migrating primitive streak for node morphogenesis and left-right patterning. Hum Mol Genet. 2013; 22:1913–1923. doi: 10.1093/hmg/ddt001 23303524 PMC3633368

[11] Li AH, Hanchard NA, Azamian M, D’Alessandro LCA, Coban-Akdemir Z, Lopez KN, Hall NJ, Dickerson H, Nicosia A, Fernbach S, et al: Genetic architecture of laterality defects revealed by whole exome sequencing. Eur J Hum Genet. 2019; 27:563–573. doi: 10.1038/s41431-018-0307-z 30622330 PMC6460585

[12] French VM, van de Laar IM, Wessels MW, Rohe C, Roos-Hesselink JW, Wang G, Frohn-Mulder IM, Severijnen LA, de Graaf BM, Schot R, et al: NPHP4 variants are associated with pleiotropic heart malformations. Circ Res. 2012; 110:1564–1574. doi: 10.1161/CIRCRESAHA.112.269795 22550138 PMC3916111

[13] Nöthe-Menchen T, Wallmeier J, Pennekamp P, Höben IM, Olbrich H, Loges NT, Raidt J, Dougherty GW, Hjeij R, Dworniczak B, Omran H: Randomization of Left-right Asymmetry and Congenital Heart Defects: The Role of DNAH5 in Humans and Mice. Circ Genom Precis Med. 2019. doi: 10.1161/CIRCGEN.119.002686 31638833 PMC7174103

[14] Zaidi S, Choi M, Wakimoto H, Ma L, Jiang J, Overton JD, Romano-Adesman A, Bjornson RD, Breitbart RE, Brown KK, et al: De novo mutations in histone-modifying genes in congenital heart disease. Nature. 2013; 498:220–223. doi: 10.1038/nature12141 23665959 PMC3706629

[15] Homsy J, Zaidi S, Shen Y, Ware JS, Samocha KE, Karczewski KJ, DePalma SR, McKean D, Wakimoto H, Gorham J, et al: De novo mutations in congenital heart disease with neurodevelopmental and other congenital anomalies. Science. 2015; 350:1262–1266. doi: 10.1126/science.aac9396 26785492 PMC4890146

[16] Krawitz PM, Schweiger MR, Rödelsperger C, Marcelis C, Kölsch U, Meisel C, Stephani F, Kinoshita T, Murakami Y, Bauer S, et al: Identity-by-descent filtering of exome sequence data identifies PIGV mutations in hyperphosphatasia mental retardation syndrome. Nat Genet. 2010; 42:827–829. doi: 10.1038/ng.653 20802478

[17] Liang S, Shi X, Yu C, Shao X, Zhou H, Li X, Chang C, Lai KS, Ma J, Zhang R: Identification of novel candidate genes in heterotaxy syndrome patients with congenital heart diseases by whole exome sequencing. Biochim Biophys Acta Mol Basis Dis. 2020; 1866:165906. doi: 10.1016/j.bbadis.2020.165906 32738303

[18] Tariq M, Belmont JW, Lalani S, Smolarek T, Ware SM: SHROOM3 is a novel candidate for heterotaxy identified by whole exome sequencing. Genome Biol. 2011; 12:R91. doi: 10.1186/gb-2011-12-9-r91 21936905 PMC3308054

[19] Nishimura DY, Baye LM, Perveen R, Searby CC, Avila-Fernandez A, Pereiro I, Ayuso C, Valverde D, Bishop PN, Manson FD et al: Discovery and functional analysis of a retinitis pigmentosa gene, C2ORF71. American journal of human genetics. 2010; 86(5):686–695. doi: 10.1016/j.ajhg.2010.03.005 20398886 PMC2868997

[20] Gabriel GC, Young CB, Lo CW: Role of cilia in the pathogenesis of congenital heart disease. Seminars in cell & developmental biology. 2021; 110:2–10. doi: 10.1016/j.semcdb.2020.04.017 32418658 PMC7906359

[21] Matsui T, Bessho Y: Left-right asymmetry in zebrafish. Cellular and molecular life sciences: CMLS. 2012; 69(18):3069–3077. 22527718 10.1007/s00018-012-0985-6 PMC11115138

[22] Borovina A, Superina S, Voskas D, Ciruna B: Vangl2 directs the posterior tilting and asymmetric localization of motile primary cilia. Nature cell biology. 2010; 12(4):407–412. doi: 10.1038/ncb2042 20305649

[23] Boskovski MT, Yuan S, Pedersen NB, Goth CK, Makova S, Clausen H, Brueckner M, Khokha MK: The heterotaxy gene GALNT11 glycosylates Notch to orchestrate cilia type and laterality. Nature. 2013; 504:456–459. doi: 10.1038/nature12723 24226769 PMC3869867

[24] Deng H, Xia H, Deng S: Genetic basis of human left-right asymmetry disorders. Expert Rev Mol Med. 2015; 16:e19. doi: 10.1017/erm.2014.22 26258520

[25] Montague TG, Gagnon JA, Schier AF: Conserved regulation of Nodal-mediated left-right patterning in zebrafish and mouse. Development. 2018; 145. doi: 10.1242/dev.171090 30446628 PMC6307886

[26] Ocaña OH, Coskun H, Minguillón C, Murawala P, Tanaka EM, Galcerán J, Muñoz-Chápuli R, Nieto MA: A right-handed signalling pathway drives heart looping in vertebrates. Nature. 2017; 549:86–90. doi: 10.1038/nature23454 28880281 PMC5590727

[27] Lin AE, Krikov S, Riehle-Colarusso T, Frías JL, Belmont J, Anderka M, Geva T, Getz KD, Botto LD: Laterality defects in the national birth defects prevention study (1998–2007): birth prevalence and descriptive epidemiology. Am J Med Genet A. 2014; 164a:2581–2591. doi: 10.1002/ajmg.a.36695 25099286 PMC4462240

[28] Richards S, Aziz N, Bale S, Bick D, Das S, Gastier-Foster J, Grody WW, Hegde M, Lyon E, Spector E, et al: Standards and guidelines for the interpretation of sequence variants: a joint consensus recommendation of the American College of Medical Genetics and Genomics and the Association for Molecular Pathology. Genet Med. 2015; 17:405–424. doi: 10.1038/gim.2015.30 25741868 PMC4544753

[29] Cangiano B, Swee DS, Quinton R, Bonomi M: Genetics of congenital hypogonadotropic hypogonadism: peculiarities and phenotype of an oligogenic disease. Hum Genet. 2021; 140:77–111. doi: 10.1007/s00439-020-02147-1 32200437

[30] Herbert A: Z-DNA and Z-RNA in human disease. Commun Biol. 2019; 2:7. doi: 10.1038/s42003-018-0237-x 30729177 PMC6323056

[31] Awad IA, Polster SP: Cavernous angiomas: deconstructing a neurosurgical disease. J Neurosurg. 2019; 131:1–13. doi: 10.3171/2019.3.JNS181724 31261134 PMC6778695

[32] Zhang Y, Chen W, Zeng W, Lu Z, Zhou X: Biallelic loss of function NEK3 mutations deacetylate α-tubulin and downregulate NUP205 that predispose individuals to cilia-related abnormal cardiac left-right patterning. Cell Death Dis. 2020; 11:1005. 33230144 10.1038/s41419-020-03214-1 PMC7684299

[33] Vetrini F, D’Alessandro LC, Akdemir ZC, Braxton A, Azamian MS, Eldomery MK, Miller K, Kois C, Sack V, Shur N, et al: Bi-allelic Mutations in PKD1L1 Are Associated with Laterality Defects in Humans. Am J Hum Genet. 2016; 99:886–893. doi: 10.1016/j.ajhg.2016.07.011 27616478 PMC5065643

[34] Wells JR, Padua MB, Ware SM: The genetic landscape of cardiovascular left-right patterning defects. *Current opinion in genetics & development*. 2022; 75:101937. doi: 10.1016/j.gde.2022.101937 35777348 PMC10698510

[35] Gokey JJ, Ji Y, Tay HG, Litts B, Amack JD: Kupffer’s vesicle size threshold for robust left-right patterning of the zebrafish embryo. *Developmental dynamics*: *an official publication of the American Association of Anatomists*. 2016; 245(1):22–33. doi: 10.1002/dvdy.24355 26442502 PMC5434515

[36] Knowles MR, Zariwala M, Leigh M: Primary Ciliary Dyskinesia. *Clinics in chest medicine*. 2016; 37(3):449–461. doi: 10.1016/j.ccm.2016.04.008 27514592 PMC4988337

[37] Barr FA: Membrane traffic: Golgi stumbles over cilia. Curr Biol. 2009; 19:R253–255. doi: 10.1016/j.cub.2009.01.049 19321142

[38] Follit JA, San Agustin JT, Xu F, Jonassen JA, Samtani R, Lo CW, Pazour GJ: The Golgin GMAP210/TRIP11 anchors IFT20 to the Golgi complex. PLoS Genet. 2008; 4:e1000315. doi: 10.1371/journal.pgen.1000315 19112494 PMC2602600

[39] Wang Z, Shi Y, Ma S, Huang Q, Yap YT, Shi L, Zhang S, Zhou T, Li W, Hu B, et al: Abnormal fertility, acrosome formation, IFT20 expression and localization in conditional Gmap210 knockout mice. Am J Physiol Cell Physiol. 2020; 318:C174–c190. doi: 10.1152/ajpcell.00517.2018 31577511 PMC6985835

[40] Yuan S, Zhao L, Brueckner M, Sun Z: Intraciliary calcium oscillations initiate vertebrate left-right asymmetry. Curr Biol. 2015; 25:556–567. doi: 10.1016/j.cub.2014.12.051 25660539 PMC4469357

[41] Tan C, Meng L, Lv M, He X, Sha Y, Tang D, Tan Y, Hu T, He W, Tu C, et al: Bi-allelic variants in DNHD1 cause flagellar axoneme defects and asthenoteratozoospermia in humans and mice. American journal of human genetics. 2022; 109(1):157–171. doi: 10.1016/j.ajhg.2021.11.022 34932939 PMC8764202

[42] Guan Y, Yang H, Yao X, Xu H, Liu H, Tang X, Hao C, Zhang X, Zhao S, Ge W, Ni X: Clinical and Genetic Spectrum of Children with Primary Ciliary Dyskinesia in China. Chest. 2021. doi: 10.1016/j.chest.2021.02.006 33577779 PMC8129725

[43] Olbrich H, Häffner K, Kispert A, Völkel A, Volz A, Sasmaz G, Reinhardt R, Hennig S, Lehrach H, Konietzko N, et al: Mutations in DNAH5 cause primary ciliary dyskinesia and randomization of left-right asymmetry. Nat Genet. 2002; 30:143–144. doi: 10.1038/ng817 11788826

[44] Fliegauf M, Olbrich H, Horvath J, Wildhaber JH, Zariwala MA, Kennedy M, Knowles MR, Omran H: Mislocalization of DNAH5 and DNAH9 in respiratory cells from patients with primary ciliary dyskinesia. Am J Respir Crit Care Med. 2005; 171:1343–1349. doi: 10.1164/rccm.200411-1583OC 15750039 PMC2718478

[45] Schwabe GC, Hoffmann K, Loges NT, Birker D, Rossier C, de Santi MM, Olbrich H, Fliegauf M, Failly M, Liebers U, et al: Primary ciliary dyskinesia associated with normal axoneme ultrastructure is caused by DNAH11 mutations. Hum Mutat. 2008; 29:289–298. doi: 10.1002/humu.20656 18022865

[46] Dong J, Wyss A, Yang J, Price TR, Nicolas A, Nalls M, Tranah G, Franceschini N, Xu Z, Schulte C, et al: Genome-Wide Association Analysis of the Sense of Smell in U.S. Older Adults: Identification of Novel Risk Loci in African-Americans and European-Americans. Mol Neurobiol. 2017; 54:8021–8032. doi: 10.1007/s12035-016-0282-8 27878761 PMC5441979

[47] McKenzie CW, Craige B, Kroeger TV, Finn R, Wyatt TA, Sisson JH, Pavlik JA, Strittmatter L, Hendricks GM, Witman GB, Lee L: CFAP54 is required for proper ciliary motility and assembly of the central pair apparatus in mice. Mol Biol Cell. 2015; 26:3140–3149. doi: 10.1091/mbc.E15-02-0121 26224312 PMC4569307

[48] Sha Y, Wei X, Ding L, Ji Z, Mei L, Huang X, Su Z, Wang W, Zhang X, Lin S: Biallelic mutations of CFAP74 may cause human primary ciliary dyskinesia and MMAF phenotype. J Hum Genet. 2020; 65:961–969. doi: 10.1038/s10038-020-0790-2 32555313

[49] Gur M, Cohen EB, Genin O, Fainsod A, Perles Z, Cinnamon Y: Roles of the cilium-associated gene CCDC11 in left-right patterning and in laterality disorders in humans. Int J Dev Biol. 2017; 61:267–276. doi: 10.1387/ijdb.160442yc 28621423

[50] Noël ES, Momenah TS, Al-Dagriri K, Al-Suwaid A, Al-Shahrani S, Jiang H, Willekers S, Oostveen YY, Chocron S, Postma AV, et al: A Zebrafish Loss-of-Function Model for Human CFAP53 Mutations Reveals Its Specific Role in Laterality Organ Function. Hum Mutat. 2016; 37:194–200. doi: 10.1002/humu.22928 26531781

[51] Narasimhan V, Hjeij R, Vij S, Loges NT, Wallmeier J, Koerner-Rettberg C, Werner C, Thamilselvam SK, Boey A, Choksi SP, et al: Mutations in CCDC11, which encodes a coiled-coil containing ciliary protein, causes situs inversus due to dysmotility of monocilia in the left-right organizer. Hum Mutat. 2015; 36:307–318. doi: 10.1002/humu.22738 25504577

[52] Fuller ZL, Berg JJ, Mostafavi H, Sella G, Przeworski M: Measuring intolerance to mutation in human genetics. Nat Genet. 2019; 51:772–776. doi: 10.1038/s41588-019-0383-1 30962618 PMC6615471

[53] Kumar S, Warrell J, Li S, McGillivray PD, Meyerson W, Salichos L, Harmanci A, Martinez-Fundichely A, Chan CWY, Nielsen MM, et al: Passenger Mutations in More Than 2,500 Cancer Genomes: Overall Molecular Functional Impact and Consequences. Cell. 2020; 180:915–927.e916. doi: 10.1016/j.cell.2020.01.032 32084333 PMC7210002

[54] Nesti C, Meschini MC, Meunier B, Sacchini M, Doccini S, Romano A, Petrillo S, Pezzini I, Seddiki N, Rubegni A, et al: Additive effect of nuclear and mitochondrial mutations in a patient with mitochondrial encephalomyopathy. Hum Mol Genet. 2015; 24:3248–3256. doi: 10.1093/hmg/ddv078 25736212

[55] Demily C, Lesca G, Poisson A, Till M, Barcia G, Chatron N, Sanlaville D, Munnich A: Additive Effect of Variably Penetrant 22q11.2 Duplication and Pathogenic Mutations in Autism Spectrum Disorder: To Which Extent Does the Tree Hide the Forest? J Autism Dev Disord. 2018; 48:2886–2889. doi: 10.1007/s10803-018-3552-7 29589274

[56] Mookerjee-Basu J, Hooper R, Gross S, Schultz B, Go CK, Samakai E, Ladner J, Nicolas E, Tian Y, Zhou B, et al: Suppression of Ca(2+) signals by EGR4 controls Th1 differentiation and anti-cancer immunity in vivo. EMBO Rep. 2020; 21:e48904. 32212315 10.15252/embr.201948904 PMC7202224

[57] MacArthur DG, Manolio TA, Dimmock DP, Rehm HL, Shendure J, Abecasis GR, Adams DR, Altman RB, Antonarakis SE, Ashley EA et al: Guidelines for investigating causality of sequence variants in human disease. *Nature*. 2014; 508(7497):469–476. doi: 10.1038/nature13127 24759409 PMC4180223

[58] Strande NT, Riggs ER, Buchanan AH, Ceyhan-Birsoy O, DiStefano M, Dwight SS, Goldstein J, Ghosh R, Seifert BA, Sneddon TP et al: Evaluating the Clinical Validity of Gene-Disease Associations: An Evidence-Based Framework Developed by the Clinical Genome Resource. *American journal of human genetics*. 2017; 100(6):895–906. doi: 10.1016/j.ajhg.2017.04.015 28552198 PMC5473734

[59] Yu PC, Gu SY, Bu JW, Du JL: TRPC1 is essential for in vivo angiogenesis in zebrafish. Circ Res. 2010; 106:1221–1232. doi: 10.1161/CIRCRESAHA.109.207670 20185799

[60] Thisse C, Thisse B, Schilling TF, Postlethwait JH: Structure of the zebrafish snail1 gene and its expression in wild-type, spadetail and no tail mutant embryos. Development. 1993; 119:1203–1215. doi: 10.1242/dev.119.4.1203 8306883

[61] Xu B, Zhang Y, Du XF, Li J, Zi HX, Bu JW, Yan Y, Han H, Du JL: Neurons secrete miR-132-containing exosomes to regulate brain vascular integrity. Cell Res. 2017; 27:882–897. doi: 10.1038/cr.2017.62 28429770 PMC5518987

[62] Sampaio P, Ferreira RR, Smith DJ, Lopes SS: Left-right organizer flow dynamics: how much cilia activity reliably yields laterality? Dev Cell. 2014; 29(6):716–728 doi: 10.1016/j.devcel.2014.04.030 24930722

