# Supplementary figures and images for: LOF variants identifying candidate genes of laterality defects patients with congenital heart disease
Source: PLoS Genet. 2022 Dec 2;18(12):e1010530. doi: 10.1371/journal.pgen.1010530 (PMC9749982; doi:10.1371/journal.pgen.1010530)

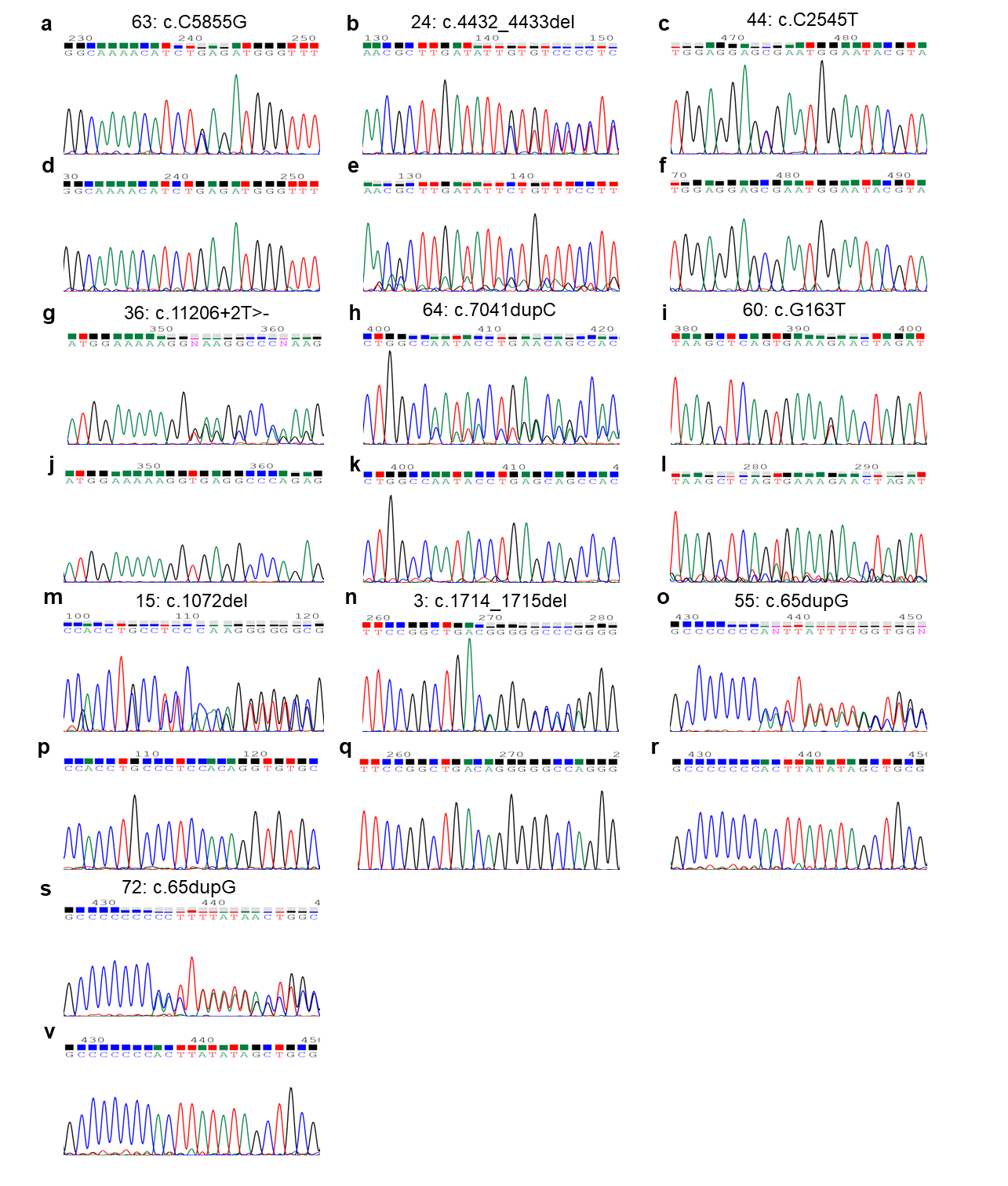

Supplement: S1 Fig — (a, b) Sanger sequencing shows frameshift or nonsense variants in TRIP11. (c, g, h) Sanger sequencing shows frameshift, splice-region mutant alleles or nonsense variants in DNHD1. (i, m, n) Sanger sequencing shows frameshift or nonsense variants in CFAP74. (o, s) Sanger sequencing shows frameshift variants in EGR4. (d, e, f, j, k, l, p, q, r, v) Sanger sequencing shows normal results that did not alter the sequences. (TIF) [file pgen.1010530.s001.tif]

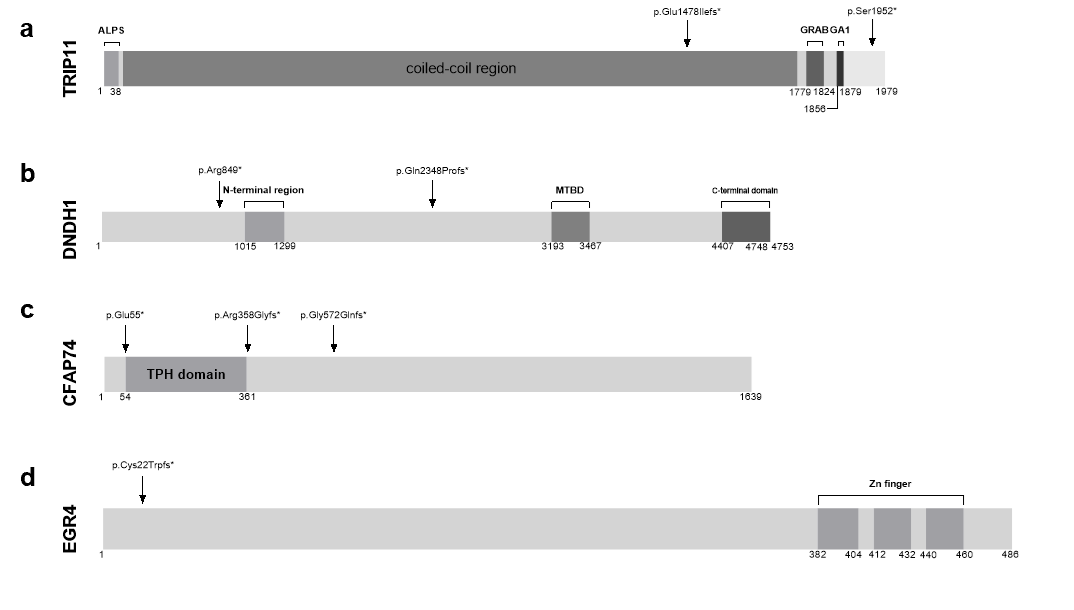

Supplement: S2 Fig — (a) the position of domains and variants in TRIP11. ALPS, ALPS (amphipathic lipid-packing sensor) motif; GRAB, GRAB (Grip-related Arf-binding) domain; GA1, GRAB-associated region. (b) the position of domains and variants in DNHD1. MTBD, microtubule-binding domain. (c) the position of domains and variants in CFAP74. TPH, Trichohyalin-plectin-homology domain. (d) the position of domains and variants in EGR4. (TIF) [file pgen.1010530.s002.tif]

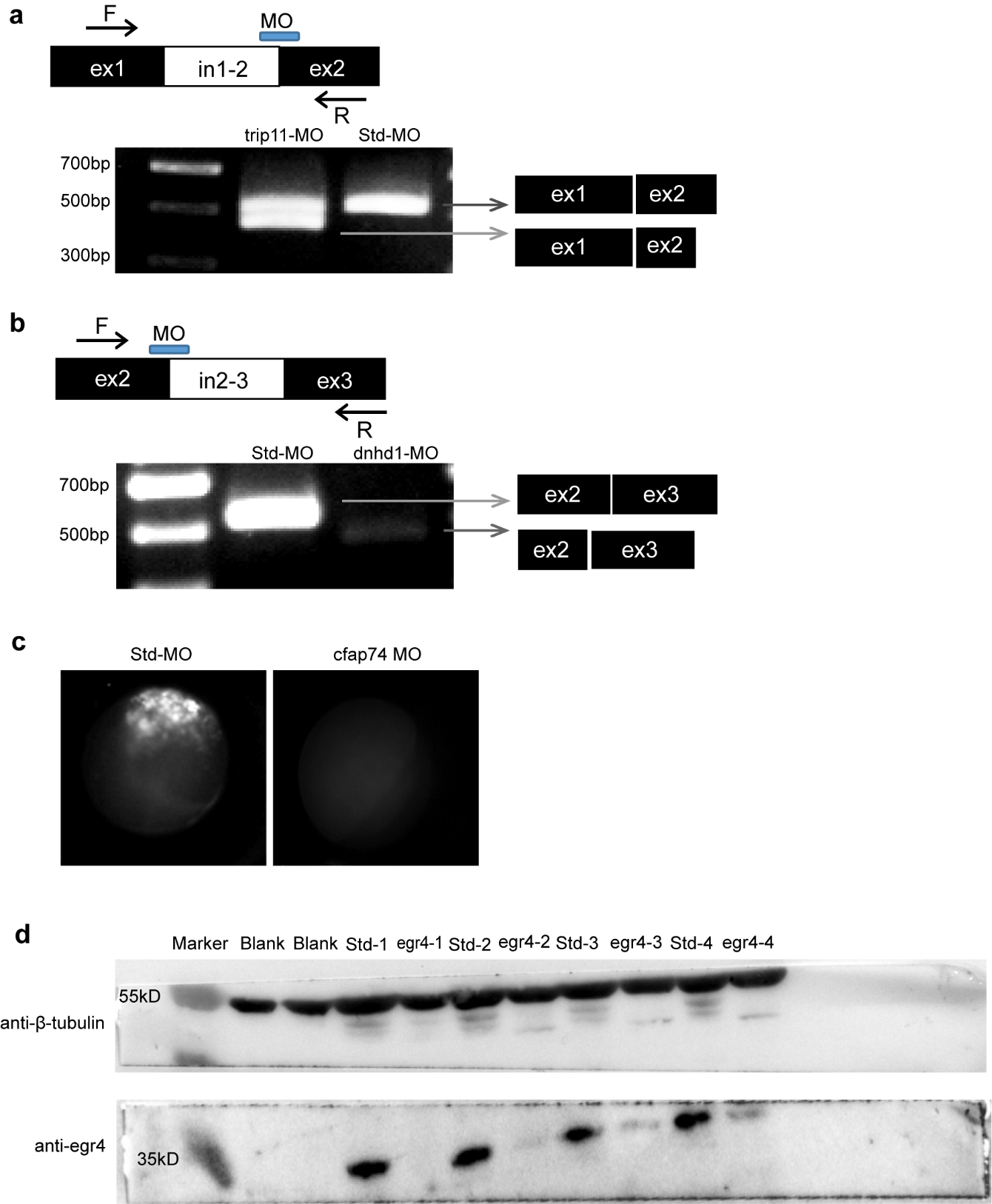

Supplement: S3 Fig — (a-b) The RT-PCR (reverse transcription-PCR) results were conducted to analyze the efficiency of sb-MOs targeting trip11, and dnhd1. Total RNA was extracted from 2 dpf zebrafish embryos. (a) The trip11 splice blocking morpholino (sb-MO) targets the junction of intron 1–2 and exon 2 resulting in a shorter exon 2. (b) The dnhd1 sb-MO target the junction of exon 2, and intron 2–3 results in a shorter exon 2. (c,d) The egr4 MO and cfap74 MO target AUG result in lower protein expression. (c) Fluorescent immunostaining of zebrafish embryo using anti-GFP antibodies in Std embryos and cfap74 morphants. The fusion gene vector and cfap74 MO or control MO were microinjected at the one-cell stage. (d) Western blot revealed knockdown of protein expression in egr4 morphants. Anti-actin was used as a loading control. Proteins were extracted from 3 dpf zebrafish embryos. Std standard control; ex, exon; in, intron. (TIF) [file pgen.1010530.s003.tif]

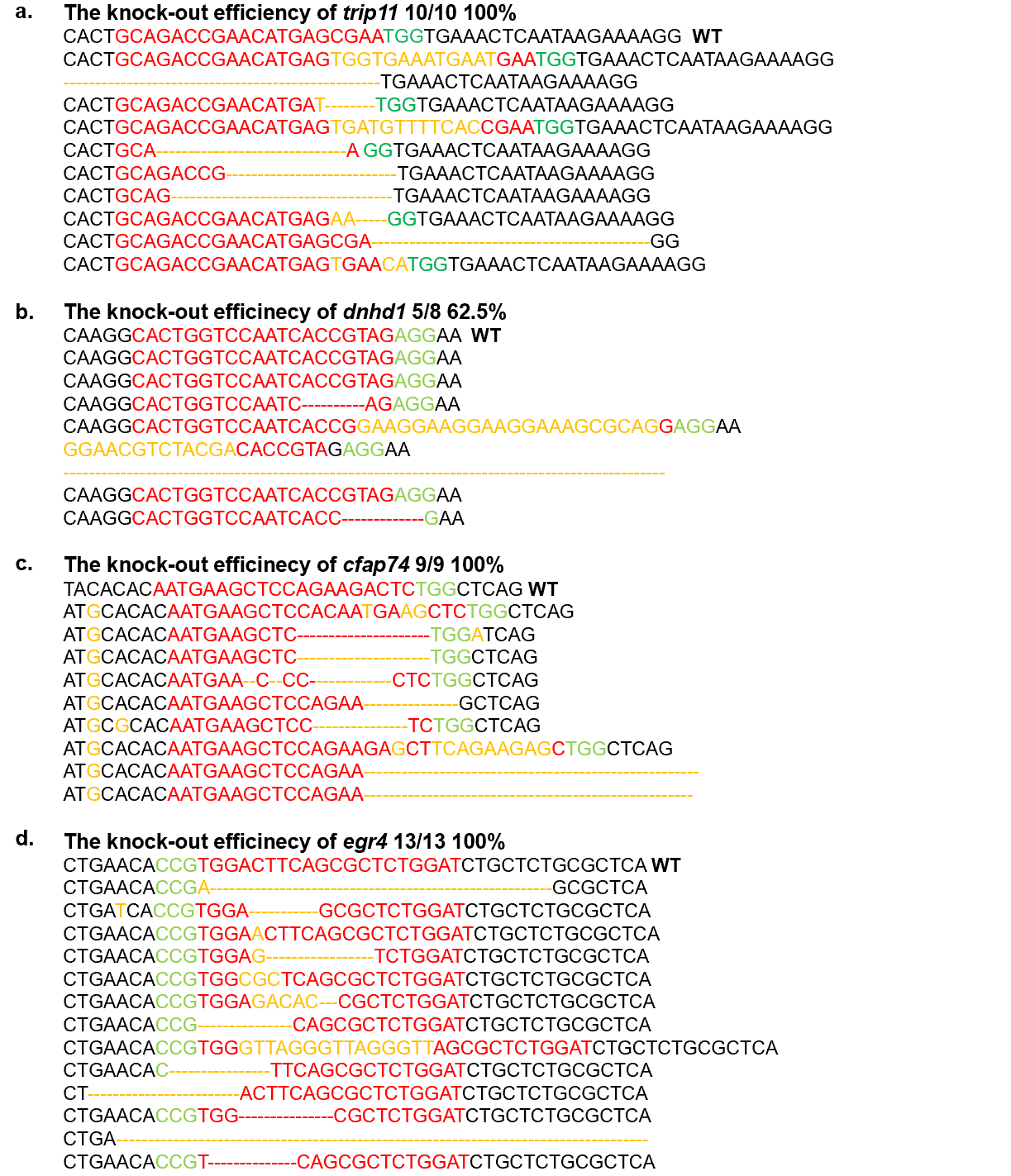

Supplement: S4 Fig — Sequence analysis of trip11, dnhd1, cfap74, and egr4 mutations caused by co-injection of zebrafish codon-optimized protein and corresponding gRNA. The red fonts show the target sites of gRNA, yellow fonts and blanks show mutated sequences, and the green fonts show the PAM sequences. (TIF) [file pgen.1010530.s004.tif]
