# Supplementary material for: LOF variants identifying candidate genes of laterality defects patients with congenital heart disease
Source: PLoS Genet. 2022 Dec 2;18(12):e1010530. doi: 10.1371/journal.pgen.1010530 (PMC9749982; doi:10.1371/journal.pgen.1010530)
Supplement: S2 Table — (DOCX) [file pgen.1010530.s006.docx]

| **Table S2 the bioinformatics information on the variants of patients with selected LOF mutations** | | | | | | | | | | | | |
| --- | --- | --- | --- | --- | --- | --- | --- | --- | --- | --- | --- | --- |
| **ID** | **Gene** | **Mutation site** | **Amino acid change** | **Exonic Function** | **SIFT score** | **SIFT pred** | **PP2 HDIV score** | **PP2 HDIV pred** | **Mutation Taster score** | **Mutation Taster pred** | **CADD raw** | **CADD pred** |
| 60 | *DNAH5* | NM_001369.3:c.12367C>T | p.His4123Tyr | nonsynonymous SNV | 0 | Del | 0.888 | Pro | 1 | Dis | 2.93 | 15.76 |

PP2: Polyphen2; pred: predicting; Del: Deleterious; Tol: tolerated; Dam: damaging; Pro: Probably damaging; Ben: benign; Dis: disease-causing; N: polymorphism.
