# Supplementary material for: LOF variants identifying candidate genes of laterality defects patients with congenital heart disease
Source: PLoS Genet. 2022 Dec 2;18(12):e1010530. doi: 10.1371/journal.pgen.1010530 (PMC9749982; doi:10.1371/journal.pgen.1010530)
Supplement: S3 Table — (DOCX) [file pgen.1010530.s007.docx]

| **Table S3 the bioinformatics information on the variants of candidate genes** | | | | | | | | | | | | |
| --- | --- | --- | --- | --- | --- | --- | --- | --- | --- | --- | --- | --- |
| **ID** | **Gene** | **Mutation site** | **Amino acid change** | **Exonic Function** | **SIFT score** | **SIFT pred** | **PP2 HDIV score** | **PP2 HDIV pred** | **Mutation Taster score** | **Mutation Taster pred** | **CADD raw** | **CADD pred** |
| 58 | *CFAP74* | NM_001080484.1:c.938C>T | p.Ala313Val | nonsynonymous SNV | 0.28 | Tol | 0.995 | Dam | 1 | N | 0.966 | 8.941 |
| 12 | *CFAP74* | NM_001080484.1:c.737G>A | p.Arg246Gln | nonsynonymous SNV | 0.81 | Tol | 0.014 | Ben | 1 | Dis | -0.538 | 1.557 |
| 1 | *DNHD1* | NM_144666.3:c.2282C>G | p.Pro761Arg | nonsynonymous SNV | 0.52 | Tol | 0.993 | Dam | 1 | N | 3.948 | 20.2 |
| 50 | *DNHD1* | NM_144666.3:c.2282C>G | p.Pro761Arg | nonsynonymous SNV | 0.52 | Tol | 0.993 | Dam | 1 | N | 3.948 | 20.2 |
| 23 | *DNHD1* | NM_144666.3:c.3574C>T | p.Arg1192Cys | nonsynonymous SNV | 0.06 | Tol | 0.993 | Dam | 1 | N | 1.727 | 11.73 |
| 6 | *DNHD1* | NM_144666.3:c.8614G>A | p.Val2872Met | nonsynonymous SNV | 0.01 | Del | 1 | Dam | 1 | N | 3.385 | 17.42 |
| 56 | *DNHD1* | NM_144666.3:c.11767G>A | p.Val3923Met | nonsynonymous SNV | 0.16 | Tol | 0.458 | Pro | 1 | N | -0.725 | 0.894 |
| 16 | *DNHD1* | NM_144666.3:c.12617G>A | p.Arg4206His | nonsynonymous SNV | 0 | Del | 1 | Dam | 1 | Dis | 4.111 | 21.2 |
| 38 | *DNHD1* | NM_144666.3:c.12617G>A | p.Arg4206His | nonsynonymous SNV | 0 | Del | 1 | Dam | 1 | Dis | 4.111 | 21.2 |
| 57 | *DNHD1* | NM_144666.3:c.620A>T | p.Glu207Val | nonsynonymous SNV | 0.03 | Del | 0.418 | Ben | 0.979 | N | 2.605 | 14.67 |

PP2: Polyphen2; pred: predicting; Del: Deleterious; Tol: tolerated; Dam: damaging; Pro: Probably damaging; Ben: benign; Dis: disease-causing; N: polymorphism.
