# Supplementary material for: LOF variants identifying candidate genes of laterality defects patients with congenital heart disease
Source: PLoS Genet. 2022 Dec 2;18(12):e1010530. doi: 10.1371/journal.pgen.1010530 (PMC9749982; doi:10.1371/journal.pgen.1010530)
Supplement: S4 Table — (DOCX) [file pgen.1010530.s008.docx]

| **Table S4 Clinical phenotypes of laterality defects patients with rare nonsynonymous variants of three candidate genes** | | | | |
| --- | --- | --- | --- | --- |
| **ID** | **Gene** | **Mutation site** | **Patients’ cardiac abnormalities** | **Extracardiac abnormalities** |
| 58 | CFAP74 | p.Ala313Val | M, ASI, PS, DORV, ASD, VSD | BI, SRS, RS, LSL |
| 12 | CFAP74 | p.Arg246Gln | L, ILAA, DORV, PS, VSD | BLB, polysplenia, RS, LCS |
| 1 | DNHD1 | p.Pro761Arg | D, IRAA, SV, DORV, CAVC, VSD | BRB, asplenia, RS, LCS |
| 50 | DNHD1 | p.Pro761Arg | L, ASI, TGA, PA, PDA, CAVC, ASD | BI, SRS, LS, LCS |
| 23 | DNHD1 | p.Arg1192Cys | D, IRAA, SV, MGA, PS, ASD, CAVC | BRB, asplenia, LS, LCS |
| 6 | DNHD1 | p.Val2872Met | D, IRAA, VSI, MGA, PS | BRB, asplenia, LS, LCS |
| 56 | DNHD1 | p.Val3923Met | D, SA, VSI, PS | BLB, polysplenia, LS, RSL |
| 16 | DNHD1 | p.Arg4206His | D, IRAA, SV, MGA, PS, ASD | BI, asplenia, NA, LCS |
| 38 | DNHD1 | p.Arg4206His | D, ASI, VSI, PA, PDA, ASD, VSD | BI, SRS, RS, LSL |
| 57 | DNHD1 | p.Glu207Val | L, ASI, SV, PS | BI, SRS, RS, LSL |

D dextrocardia, M mesocardia, ASI atrial situs inversus, IRAA isomerism of right atrial appendages, ILAA isomerism of left atrial appendages, VSS ventricular situs solitus, VSI ventricular situs inversus, SV single ventricle, PA pulmonary atresia, PS pulmonary stenosis, MGA malposed great arteries, TGA transposed great arteries, DORV double outlet right ventricle, PDA patent ductus arteriosus, CAVC complete atrioventricular canal, ASD atrial septum defect, VSD ventricle septum defect, BI bronchial inversus, BRB bilateral right bronchi (short), BLB bilateral left bronchi (long), SRS single right spleen, SLS single left spleen, RS right-sided stomach, LS left-sided stomach, SCS stomach centrally situated, LSL left-sided liver, RSL right-sided liver, LCS liver centrally situated, NA not available.
