# Supplementary material for: LOF variants identifying candidate genes of laterality defects patients with congenital heart disease
Source: PLoS Genet. 2022 Dec 2;18(12):e1010530. doi: 10.1371/journal.pgen.1010530 (PMC9749982; doi:10.1371/journal.pgen.1010530)
Supplement: S5 Table — (DOCX) [file pgen.1010530.s009.docx]

| **Table S5 the primers of sanger sequencing** | | |
| --- | --- | --- |
| **ID** | **Gene** | **primer** |
| 63 | *TRIP11* | F: 5'-AATATGCCTCCAATGGCCAACT-3' |
|  |  | R: 5'-AGCAAACCCATGTTTAACTGAGA-3' |
| 24 | *TRIP11* | F: 5'-AATCCCCACCGCACTAAGAC-3' |
|  |  | R: 5'-AAGCAAGGCGAAAATGGTGTC-3' |
| 44 | *DNHD1* | F: 5'-TTGGAGCCACCATCTGGAAC-3' |
|  |  | R: 5'-TATCAAGACTGGCCCAGGGA-3' |
| 36 | *DNHD1* | F: 5'-CTTCTCTCGCAGTGTGGGAT-3' |
|  |  | R: 5'-TGAACATGATTCACGGTTTCTTCTT-3' |
| 64 | *DNHD1* | F: 5'-CAACATAGCCTACCTCCGCA-3' |
|  |  | R: 5'-AGCTGATTTCCGTGAAGCCT-3' |
| 60 | *CFAP74* | F: 5'-GGGCCCATCCTGCTCAAAAG-3' |
|  |  | R: 5'-CCATAAAGGGACCGGAGAGG-3' |
| 15 | *CFAP74* | F: 5'-CCGTAGTGTGTCTTGTCCCCTT-3' |
|  |  | R: 5'-CAGGGGACCTCTTGCACTCA-3' |
| 3 | *CFAP74* | F: 5'-CTGGCCCCTTTTTGACTTGC-3' |
|  |  | R: 5'-GCCAGGGGGTAGAGTAGACA-3' |
| 55 | *EGR4* | F: 5'-CTAAGCTTCCCATCGCCCC-3' |
|  |  | R: 5'-CGTTAAGGGGGACTGAGTGTC-3' |
| 72 | *EGR4* | F: 5'-ACACTTGCTCCTGACACCTG-3' |
|  |  | R: 5'-TGACTTCTTTGTGAAAGATAGGAGA-3' |
