# Supplementary material for: LOF variants identifying candidate genes of laterality defects patients with congenital heart disease
Source: PLoS Genet. 2022 Dec 2;18(12):e1010530. doi: 10.1371/journal.pgen.1010530 (PMC9749982; doi:10.1371/journal.pgen.1010530)
Supplement: S6 Table — (DOCX) [file pgen.1010530.s010.docx]

| **Table S6 Antisense RNA probes conducted for whole mount in situs hybridization** | | | | |
| --- | --- | --- | --- | --- |
| **Gene** | **primer** | **location** | **length(bp)** | **vector** |
| *trip11* | - | Chr13: 33386745-33393441 | 1118 | pGEM-T Easy |
|  | - |  |  |  |
| *dnhd1* | - | Chr11: 13104847-13108752 | 1133 | pGEM-T Easy |
|  | - |  |  |  |
| *cfap74* | - | Chr8: 54228695-54237950 | 744 | pGEM-T Easy |
|  | - |  |  |  |
| *egr4* | - | Chr23: 46581594-46582941 | 1180 | pGEM-T Easy |
|  | - |  |  |  |
| *spaw* | F: 5'-CACCCTCAGACGAGACACGG-3' | Chr5: 68524501-68526905 | 1259 | pGEM-T Easy |
|  | R: 5'-CACTCCTCCACGATCATGTCC-3' |  |  |  |
| *lefty2* | F: 5'-TCAGCGTCTTGTGTTCG-3' | Chr17: 8308293-8311008 | 872 | pGEM-T Easy |
|  | R: 5'-TCAGTGGGGATTTGGGG-3' |  |  |  |
| *pitx2* | F: 5'-TCTCCTTGCTCTCGGCT-3' | Chr14: 36226327-36229189 | 1288 | pGEM-T Easy |
|  | R: 5'-CTTGTTCCTGGGATTCG-3' |  |  |  |
