# Supplementary material for: LOF variants identifying candidate genes of laterality defects patients with congenital heart disease
Source: PLoS Genet. 2022 Dec 2;18(12):e1010530. doi: 10.1371/journal.pgen.1010530 (PMC9749982; doi:10.1371/journal.pgen.1010530)
Supplement: S7 Table — (DOCX) [file pgen.1010530.s011.docx]

| **Table S7 MO sequences, injection doses, and total embryo numbers analyzed for heart looping and gene expression** | | | | | | | | | | |
| --- | --- | --- | --- | --- | --- | --- | --- | --- | --- | --- |
| **Gene** | **MO sequence 5'-3'** | **MO's function** | **Blocking site** | **Knockdown efficacy analysis** | **Dose (ng) for heart looping analysis** | **Numbers for heart looping analysis** | **Dose (ng) for gene expression analysis** | **Numbers for pitx2 expression analysis** | **Numbers for lefty2 expression analysis** | **Numbers for spaw expression analysis** |
| *trip11* | ATCTGCAAGGAAAGACATGCTGAGT | splice blocking |  | reverse transcription PCR | 4 | 373 | 4 | 496 | 426 | 632 |
| *dnhd1* | ACATCCACAATCACTTACCCTGTAT | splice blocking |  | reverse transcription PCR | 4 | 498 | 4 | 340 | 565 | 305 |
| *cfap74* | ATATTCGACACATCCATACCTCCAC | translation blocking | ATG | immuno- fluorescence | 16 | 295 | 16 | 223 | 355 | 320 |
| *egr4* | AGATGAGAATCACGCACATGGTTGA | translation blocking | ATG | western blot | 2 | 268 | 2 | 375 | 369 | 331 |
| Standard control | CCTCTTACCTCAGTTACAATTTATA | — | — | — | 16 | 296 | 16 | 300 | 370 | 233 |
| Standard control | CCTCTTACCTCAGTTACAATTTATA | — | — | — | 2 | 308 | 2 | 276 | 321 | 296 |
