# Supplementary material for: LOF variants identifying candidate genes of laterality defects patients with congenital heart disease
Source: PLoS Genet. 2022 Dec 2;18(12):e1010530. doi: 10.1371/journal.pgen.1010530 (PMC9749982; doi:10.1371/journal.pgen.1010530)
Supplement: S8 Table — (DOCX) [file pgen.1010530.s012.docx]

| **Table S8 the primers of MOs' effectiveness evaluation** | |
| --- | --- |
| **Gene** | **primer** |
| *trip11* | F: 5'-ATGGATTATATGTGTTTCTGAAAGG-3' |
|  | R: 5'-AGTAGTGGTAGTAGCAGCAGTGGTG-3' |
| *dnhd1* | F: 5'-ATTGGTGAAGCATATTGAACGAGCA-3' |
|  | R: 5'-GGGCATTGTAAAGGGATGTGGTGAG-3' |
