# Supplementary material for: LOF variants identifying candidate genes of laterality defects patients with congenital heart disease
Source: PLoS Genet. 2022 Dec 2;18(12):e1010530. doi: 10.1371/journal.pgen.1010530 (PMC9749982; doi:10.1371/journal.pgen.1010530)
Supplement: S9 Table — (DOCX) [file pgen.1010530.s013.docx]

| **Table S9 the sequences of gRNA** | |
| --- | --- |
| **Gene** | **gRNA sequence 5'-3'** |
| *trip11* | GCAGACCGAACATGAGCGAATGG |
| *dnhd1* | CACTGGTCCAATCACCGTAGAGG |
| *cfap74* | AATGAAGCTCCAGAAGACTCTGG |
| *egr4* | ATCCAGAGCGCTGAAGTCCACGG |
