# Supplementary material for: LOF variants identifying candidate genes of laterality defects patients with congenital heart disease
Source: PLoS Genet. 2022 Dec 2;18(12):e1010530. doi: 10.1371/journal.pgen.1010530 (PMC9749982; doi:10.1371/journal.pgen.1010530)
Supplement: S10 Table — (DOCX) [file pgen.1010530.s014.docx]

| **Table S10 the primers of CRISPR/Cas9-mediated gene editing' effectiveness evaluation** | |
| --- | --- |
| **Gene** | **primer** |
| *trip11* | F: 5'-CGAGAAAGACACCCGTATTGA-3' |
|  | R: 5'-CCAGCTGCTTGGTTAGACAGAGGT-3' |
| *dnhd1* | F: 5'-CCCTGCTTGTTCCAGAGCCAT-3' |
|  | R: 5'-CCTAGGAGAGTCAATGACAGGTCA-3' |
| *cfap74* | F: 5'-GGGAAGTCAGTGATGGGGAA-3' |
|  | R: 5'-GGCTGTTTATTAAAGGGGAGGAG-3' |
| *egr4* | F: 5'-TCCTGCCGACGTAGATGAGC-3' |
|  | R: 5'-GGACCGAGTGGAGAAAGCAG-3' |
