# Supplementary material for: LOF variants identifying candidate genes of laterality defects patients with congenital heart disease
Source: PLoS Genet. 2022 Dec 2;18(12):e1010530. doi: 10.1371/journal.pgen.1010530 (PMC9749982; doi:10.1371/journal.pgen.1010530)
Supplement: S11 Table — (DOCX) [file pgen.1010530.s015.docx]

| **Table S11 MO and mRNA injection doses, and total embryo numbers analyzed for rescue** | | | |
| --- | --- | --- | --- |
| **Gene** | **MO Dose (ng) for rescue analysis** | **mRNA Dose (pg) for rescue analysis** | **Numbers for rescue analysis** |
| *trip11* | 4 | 75 | 276 |
| *dnhd1* | 4 | 75 | 321 |
| *cfap74* | 16 | 100 | 251 |
| Standard control | 16 | - | 296 |
