# Supplementary material for: LOF variants identifying candidate genes of laterality defects patients with congenital heart disease
Source: PLoS Genet. 2022 Dec 2;18(12):e1010530. doi: 10.1371/journal.pgen.1010530 (PMC9749982; doi:10.1371/journal.pgen.1010530)
Supplement: S12 Table — (DOCX) [file pgen.1010530.s016.docx]

| **Table S12 Total number of KV and cilia for cilia length and CBF** | | | | |
| --- | --- | --- | --- | --- |
| **Gene** | **KV numbers for cilia length analysis** | **cilia numbers for cilia length analysis** | **KV numbers for CBF analysis** | **cilia numbers for CBF analysis** |
| *trip11* | *9* | *218* | *14* | *53* |
| *dnhd1* | *7* | *148* | *18* | *129* |
| *cfap74* | *11* | *271* | *12* | *147* |
| Standard control | 9 | 290 | 13 | 143 |
