# Supplementary material for: LOF variants identifying candidate genes of laterality defects patients with congenital heart disease
Source: PLoS Genet. 2022 Dec 2;18(12):e1010530. doi: 10.1371/journal.pgen.1010530 (PMC9749982; doi:10.1371/journal.pgen.1010530)
Supplement: Attachment — Submitted filename: reply to reviewer4.docx [file pgen.1010530.s023.docx]

Reviewer4

This is a manuscript describing exome sequencing results on a group of children affected with heterotaxy including complex congenital heart defects. This is a well-known class of birth defects with difficult genetic contributions. Some patients have Mendelian inheritance but in many others it has not been possible to find genetic causes. This paper could contribute to the literature by identifying several new candidate genes. Strengths of the work include the use of exome sequencing to survey most of the known human coding sequence and the use of zebrafish models to evaluate a highly selected group of genes that may play a role in patterning. Weaknesses include the modest sample size, the lack of information about inheritance in each case, and the lack of structured summary of evidence for a causal role for each gene and variant.

Thank you for your suggestions on this article. We have revised the article. The specific replies are as follows:

Critique:
1. The methods for annotation of the gene variants are not current. The investigators should include information from gnomAD not just for variant allele frequency but also for LoF tolerance measures. REVEL scores are also readily available from public resources and should be included with the other deleteriousness metrics like the CADD scores.

Thank you for your suggestion. The pLOF tolerance measures of gnomAD, REVEL scores, and CADD scores have been supplemented in manuscript Table 3.

2. Unfortunately parent samples are not included in the analysis and so it is not possible to evaluate for de novo occurrence. If the variants were transmitted from unaffected parents it would have also been useful to verify that they did not have milder phenotypes.

Thank you for your question. We agree that genetic information about parents is important and can improve the theories and methods of the study. Unfortunately, the clinical samples were collected years ago and the parental samples are not available. It is a pity that whether these variants are de novo cannot be determined.

3. The investigators have not used MacArthur et al (Guidelines for investigating causality of sequence variants in human disease Nature. 2014 Apr 24;508(7497):469-76. doi: 10.1038/nature13127.) or the ClinGen gene curation framework (Am J Hum Genet. 2017 Jun 1;100(6):895-906. doi: 10.1016/j.ajhg.2017.04.015) to critically evaluate the evidence that they have accumulated in this study.

Thank you for your suggestion. The guideline is really helpful to evaluate our studies. Because our sample capacity is insufficient for burden analysis to identify contributing variants and genes in control-matched cohorts, we screened rare LOF variants with high allele frequency in 70 laterality defect patients to increase the probability of identifying disease-causing genes. To verify the relationship between candidate genes and disease, we provided experimental evidence from two categories. First, three candidate genes were expressed in tissues relevant to laterality defects. Second, disruption of the candidate genes in zebrafish resulted in phenotypes similar to laterality defects. However, according to the guidelines, we do not have sufficient experimental and informatic support that the variants in the three genes are causal for laterality defects.[1, 2] This is the limitation of our study. *TRIP11*, *DNHD1*, and *CFAP74* are more likely to be candidates with new roles in left-right patterning. Meanwhile, *TRIP11* and *CFAP74* are associated with laterality defects for the first time. We provide preliminary evidence for their potential pathogenicity in laterality defects, and they are hoped to be further confirmed in larger groups of patients in the future. (Manuscript Line 370-376)

4. It would be helpful to include a decision tree or similar graphical representation of the gene filtering strategy described in the Results. It is currently hard to follow how the focus was narrowed to the four genes analyzed in the zebrafish model.

I am sorry for the unclear description and thank you for the suggestion. Previously, we misused the phrase “filter out” to mean “filter and keep.” We sincerely apologize for the confusion, and all the mistakes have been corrected.

The methodology for identifying four candidate genes is described below and shown in Figure R1. (Manuscript Line 127-155, Manuscript Figure 1)

(1) To identify the genetic causes of the laterality defects, we performed ES on 70 patients and 100 healthy individuals. The results revealed approximately 74,000 SNVs and 14,000 indels per individual.
(2) To identify potential disease-related genes in patients with laterality defects, variants were selected based on the following criteria: a. located in exon or splicing region; b. exclude synonymous variants; c. exclude variants with allele frequency >0.1% in 1000 Genomes Project or ExAC; d. absent in the dataset of 100 healthy control individuals; e. predicted to be disease-causing by at least one online program. According to these criteria, we identified 10226 rare variants, including both SNV and indel.
(3) We selected LOF variants consisting of frameshift, nonsense, and splice-site variants, and further screened 827 candidate variants.
(4) 39 genes were identified more than once in the 827 selected variants, and they did not include any known LR pattern-related genes. We then examined the functions of the 39 candidate genes to correlate the laterality defect phenotypes to specific genes. All candidate genes were identified according to the following criteria: a. participation in ciliary structure or function; b. association with LR axis formation-associated signaling pathways, including Notch, Nodal, TGF-β, Hedgehog, and Wnt; c. member of the ubiquitin ligase E3 family. We then identified 10 genes (*C11orf94*, *C16orf71*, *C2orf71*, *CABS1*, *CFAP74*, *CUL7*, *DNHD1*, *EGR4*, *FSIP2*, and *TRIP11*) related to LR patterning. Among these, *C2orf71* was excluded, because a previous study conducted a morphological examination of the *C2orf71* morphants revealed no gross defects in the body axis in zebrafish.[3]
(5) Considering gene homology, *C11orf94*, *C16orf71*, *ABS1*, *CUL7*, and *FSIP2* have no homologous genes in zebrafish. Thus, we finally choose *TRIP11*, *DNHD1*, *CFAP74*, and *EGR4* as our candidate genes.

Figure R1. Schematic of the methodology applied to identify candidate genes.

5. The cases with other deleterious variants (beginning line 240 page 17) are not clearly described. The text says four genes but only three are listed. The total number of cases where there is a candidate deleterious variant among all 70 cases is not clear. The phenotypes of these cases is not clear.

I am sorry for the unclear description. Manuscript Table 3 displays 10 **LOF** **variants** located in four candidate genes that were identified according to the methodology shown in Figure 1. The phenotypes of these cases are shown in Manuscript Table4.

Manuscript Table S2 shows the **rare** **nonsynonymous variants** of four candidate genes that were identified in 70 laterality defect patients and predicted to be potentially pathogenic by online programs. (Manuscript Line 254-262) The clinical phenotypes of cases with nonsynonymous variants were supplemented in Manuscript Table S3.

Reference:

1.	MacArthur DG, Manolio TA, Dimmock DP, Rehm HL, Shendure J, Abecasis GR, Adams DR, Altman RB, Antonarakis SE, Ashley EA *et al*: Guidelines for investigating causality of sequence variants in human disease. *Nature.* 2014; 508(7497):469-476.

2.	Strande NT, Riggs ER, Buchanan AH, Ceyhan-Birsoy O, DiStefano M, Dwight SS, Goldstein J, Ghosh R, Seifert BA, Sneddon TP *et al*: Evaluating the Clinical Validity of Gene-Disease Associations: An Evidence-Based Framework Developed by the Clinical Genome Resource. *American journal of human genetics.* 2017; 100(6):895-906.

3.	Nishimura DY, Baye LM, Perveen R, Searby CC, Avila-Fernandez A, Pereiro I, Ayuso C, Valverde D, Bishop PN, Manson FD *et al*: Discovery and functional analysis of a retinitis pigmentosa gene, C2ORF71. *American journal of human genetics.* 2010; 86(5):686-695.
