# Supplementary material for: LOF variants identifying candidate genes of laterality defects patients with congenital heart disease
Source: PLoS Genet. 2022 Dec 2;18(12):e1010530. doi: 10.1371/journal.pgen.1010530 (PMC9749982; doi:10.1371/journal.pgen.1010530)
Supplement: Attachment — Submitted filename: reply to reviewer1.docx [file pgen.1010530.s024.docx]

Dear Editor,

Thank you for allowing us to revise our manuscript. We would like to submit our revisions to the manuscript titled “LOF variants identifying candidate genes of laterality defects patients with congenital heart disease”.

We have read the comments carefully and made corresponding revisions marked red on the paper. The code of raw data will be added after received. Besides, we regret that we identified a mistake in manuscript during this revision. There supposed to be 776, rather than 827 LOF variants identified as candidates to find potential disease-related genes. According to our screening criteria, variants should be absent in the dataset of 100 healthy control individuals. 827 variants are the total number of variants before this exclusion (Supplementary table R1). We are really sorry about this clerical error and have already revised our article in this edition. We hope the revised version will meet the publication standards of Plos Genetics.

Sincerely,

Rang Xu

Responses to the reviewers’ comments:

Reviewer 1:

The authors have considerably improved the manuscript. It is now possible to follow the choices that ended with 4 genes being selected.
I am mostly satisfied by their answers to my comments.

Thank you for your suggestions on this article. We have revised and polished the article. Besides, we regret that we identified a clerical error in manuscript during preparing the dataset of 827 LOF variants. There supposed to be 776, rather than 827 LOF variants identified as candidates to find potential disease-related genes. 827 variants are the total number of LOF variants before excluding the same variants as the 100 healthy control datasets (Supplementary table R1). The bioinformatics information of 776 candidate LOF variants are listed in Manuscript supplementary table 1. (Manuscript Line 30, 136, 137)

Major comment:
Major comment 6 on CRISPR: I understand that knockout efficiency can be assessed by sequencing, but I was asking for gene expression, which the authors already do in Fig.S3 to assess the MOs effectiveness. I would insist on seeing those results, especially since the F0 show a much-reduced effect than the MOs, they are closer to the MO+mRNA results (It would maybe make sense to put all the graphs with the same y-axis range so they can be compared properly).
If the authors did not keep any of the samples from the F0, then it would be unreasonable to redo the experiments, especially as F0 would potentially differ.

Thank you for your suggestion. We co-injected Cas9 protein and gRNA into zebrafish embryos at the one-cell stage to generate F0 embryos and observed their phenotypes. Due to the delay of Cas9 translation and folding in dividing cells of the developing organism, F0 animal models are mosaics with multiple, independent genetic lesions.^1,2^ Genetic mosaicism between mutant and wild-type cells can complicate gene expression analysis. However, the F0 mutant models have the potential to approximate the stable knockout models.^3,4^

CRISPR induced smaller cytotoxicity resulting in fewer abnormal phenotypes in the negative control (7.2–9.8%) compared to morpholino (0–3.9%). Meanwhile, CRISPR mutants of *trip11*, *dnhd1*, and *cfap74* exhibited significantly increased rates of abnormal phenotypes in comparison with negative control.

According to the sequencing analysis of the CRISPR results, it is unlikely that we would detect the short deletion of mRNA transcripts by RT-PCR as we did in the effectiveness evaluation of the MOs. Meanwhile, real-time PCR may be not available to verify the expression level of CRISPR mutants.^4^ We believe WB validation is sufficient to test the expression level of target proteins in CRISPR mutants, but so far we are unable to find specific antibodies of trip11, dnhd1, and cfap74. We regret not being able to provide this result.

Minor comments

1. Line 175-176 I am not convinced by the egr4 ubiquitous expression claim at 24hpf, it seems localized mostly in the brain. Although it is consistent with the lack of effect of egr4. However, this is not central to the conclusions.

I am sorry for the error. egr4 exhibited ubiquitous expression patterns at 12 hpf, whereas it was predominantly expressed in brain at 24hpf. (Manuscript Line 175-176)

2. Line 147. The authors should explicitly state the lack of homolog gene in zebrafish.

Thank you for your suggestion. It has been revised. (Manuscript Line 147)

3. Figure 1. lacks a legend, most of it is self-explanatory, but it would help to guide the reader through the figure.

Thank you for your suggestion. The legend has been added. (Manuscript Line 708-712)

4. Figure 3. Titles over the different subpanels would clarify how each of the graphs are different. (3C) The typesetting of the MO+mRNA should be fixed. 3D should have a similar y axis as 3B and 3C to ensure proper comparison.

Thank you for your suggestion. The plots have been revised. (Manuscript Figure 3)

5. The multiple comparison adjustment used in the tests should be indicated either in the methods or figure legend.

Thank you for pointing this out. It has been added. (Manuscript Line 500-501)

Reference:

1.	Shah AN, Davey CF, Whitebirch AC, Miller AC, Moens CB. Rapid reverse genetic screening using CRISPR in zebrafish. *Nature methods* 2015; **12**(6): 535-40.

2.	Hwang WY, Fu Y, Reyon D, et al. Efficient genome editing in zebrafish using a CRISPR-Cas system. *Nature biotechnology* 2013; **31**(3): 227-9.

3.	Jao LE, Wente SR, Chen W. Efficient multiplex biallelic zebrafish genome editing using a CRISPR nuclease system. *Proceedings of the National Academy of Sciences of the United States of America* 2013; **110**(34): 13904-9.

4.	Buglo E, Sarmiento E, Martuscelli NB, et al. Genetic compensation in a stable slc25a46 mutant zebrafish: A case for using F0 CRISPR mutagenesis to study phenotypes caused by inherited disease. *PloS one* 2020; **15**(3): e0230566.

Supporting information

**Table R1.** The bioinformatics information of 827 candidate LOF variants.
