# Supplementary material for: LOF variants identifying candidate genes of laterality defects patients with congenital heart disease
Source: PLoS Genet. 2022 Dec 2;18(12):e1010530. doi: 10.1371/journal.pgen.1010530 (PMC9749982; doi:10.1371/journal.pgen.1010530)
Supplement: Attachment — Submitted filename: Reviewer comments.pdf [file pgen.1010530.s022.pdf]

Title: LOF variants identifying candidate genes of laterality defects patients with congenital heart disease

In this manuscript the authors identify new candidate genes, TRIP11, CFAP74, and DNDH1, as causes of laterality defects. The approach is mostly sound, and the writing is clear, with an easy-to-follow structure. The results are promising and convincing in most instances, but I have several comments.

There are several mistakes, omissions, and shortcuts, in the text that would benefit from an editing effort prior to revision. The methods are not always well described, and some experiments are hard to follow, or it is unclear what they bring to the story.

Furthermore, there are missing bits of information that are needed to fully assess the results and force the reader to guess at what was meant. How the four genes were chosen is not clear at all, and the strange inclusion of ITGB6 in two instance begs the question of how much post hoc rationalization was done to end with those 4 genes.

Overall, it is a great example of the power of zebrafish to test the biology of human diseases, but there are several limitations that need to be addressed.

Major comments:

The description of the identification of candidate genes is lacking, for example, the gene homology supplementary file gives no indication of why those 4 genes were selected amongst the 10 candidates. Why did the authors first filter by type of variants (line 132) instead of their functional criteria from lines 137-140? These latter criteria seem more interesting.

The 10 candidates mentioned on line 140 are never listed, it would also be useful to know to which of the three relevant criteria (lines 137-139) these genes fulfill. For example, why was C2orf71, supposedly a cilium actin regulator, not included?

Line 168-9, I am not sure I follow how the expression patterns point towards a role in LR patterning and cardiovascular development as none are expressed in the heart for example. If the authors could clarify how the expression patterns they observed match a role in LR patterning? I assume it is because all of these could be signs of cilia involvement, with the tail bud linked to the KF, but this should be explicitly stated.

Morpholinos experiment: How were the doses decided for the different genes? Morpholinos can have unspecific effect and careful titration is important, the rescue by mRNA helps with convincing these are real effects though.

Based on fig. S3 there is still a significant amount of egr4 protein in the embryos, that could explain the lack of results for this gene. I would be interested in the results of a CRISPR experiment with that gene.

CRISPR experiments: It is known that CRISPR can give rise to compensatory mechanisms. As the authors have the tools to validate the expression levels of their candidate genes, I would like to see RT-PCR or WB validations of their CRISPR knock-outs.

<u>Candidate genes exhibit global effects on early signaling pathways in LR development</u>: As the authors show that the KF is disrupted in their three candidate genes (with two of them having been selected for their function in cilia). It is not surprising to see these results as all these genes depend on the KF. I am unsure if they bring anything to the story.

Line 240-247. I may be mistaken, but this looks to be the exact methodology applied to identify those four candidate genes as described in line 127-129. And yet only two out of four (three?) genes have hits in Table S2, although comparing it to Table 3 and 4, it seems like these are nonsynonymous vs frame shift, deletion, etc… ? Could the authors comment and clarify on the differences in methodology, the point of this section is unclear.

Minor comments:

Short title: " … candidate …"

The supplementary files should include the list of variants, not only the 39 candidates, but the 827 variants at minimum.

The authors start with whole-exome sequencing (WES), before switching to Exome sequencing (ES), please stay consistent

ITGB6 is only mentioned in the submission abstract and the legend of fig S3, this should be fixed. It is also not in the list of 39 candidate genes, so it is unclear how it ended up in this manuscript

The list of genes varies between abstract and summary, authors should make the list of claims consistent.

Line 69, how much is the increase in morbidity and mortality?

Line 87, what fraction of defects are already explained by known mutations, this is important to judge the claims present in the introduction and abstract.

Line 96, '…by further experiments in animal models…'

In table 2, it is unclear what Ortholog with zebrafish corresponds to?

Unless I missed it, table 3 and 4 are never discussed or introduced.

Line 176, what is the standard MO? Need more information

Line 189-191, why is a range of percentage provided for each gene when Fig.2D shows only a single value with no error bar?

Line 210-216, why is a range of percentage provided for each gene when Fig.3 shows only a single value with no error bar?

Line 241, three or four candidate genes?

Line 257, were other cilia tested than the KF?

Line 299, DNHD1 has been linked to infertility and asthenoteratozoospermia (PMID 34932939)

Line 308, '…loss of dnhd1…'

The tables in supplementary should be in the proper order (S1 – S9)

It would help the reader if the methods followed to order of the results.

Fig.1 We assume the tail bud are red arrows

Fig.2C Are the rescue significantly different from the std MO?

Fig.4A only Normal and small are shown, an arrowhead to point the KV would be useful.

Fig.S3. The full WB should be shown and not just cropped bands.

Were the p-values adjusted for multiple comparisons in the different tests? If not, they ought to be

The legends say the experiments were repeated 3 times with at least 50 embryos in each group, but were the Chi tests performed on the pooled data?
